# Atractylodis Macrocephalae Rhizoma ameliorates diarrhea induced by cold drinks and a high-fat diet by remodeling gut microecology and restoring barrier function

**DOI:** 10.1186/s13020-026-01467-0

**Published:** 2026-07-15

**Authors:** Yajuan Tian, Chenxing Wang, Yuzhong Du, Jiayi Zheng, Su Gao, Hengpu Zhou, Meiqiu Yan, Jingjing Yu, Bai Tao, Zengguang Gao, Rui Wu, Jie Su, Suhong Chen, Guiyuan Lv

**Affiliations:** College of Pharmaceutical Sciences, Zhejiang Chinese Medical University, Hangzhou, 310053 Zhejiang China

**Keywords:** Atractylodis Macrocephalae Rhizoma, Cold drinks and a high-fat diet, Diarrhea, Intestinal microbiota dysbiosis, Intestinal barrier impairment

## Abstract

**Background:**

*Atractylodis Macrocephalae Rhizoma* (AMR) has traditionally been utilized for treating spleen deficiency diarrhea. Nevertheless, the effects and mechanisms of AMR on diarrhea caused by the consumption of cold drinks and a high-fat diet (CDHFD) remain insufficiently understood.

**Aim:**

This study aimed to explore the therapeutic effects and mechanisms of AMR in treating CDHFD-induced diarrhea.

**Methods:**

AMR was prepared as an aqueous extract, and its chemical composition was analyzed using UPLC-ESI-MS. A diarrhea model was established in ICR mice by exposure to CDHFD for four weeks, with AMR (low/high doses) administered concurrently via oral gavage. Bowel movements were evaluated using indicators such as fecal water content. Systemic inflammation was assessed by measuring pro-inflammatory cytokines via ELISA and performing peripheral blood cell counts. Intestinal barrier integrity was examined via H&E, AB-PAS staining, and immunofluorescence of tight junction proteins. Gut microbiota profiling was performed using 16S rDNA sequencing. Serum lipopolysaccharide (LPS) levels were measured via ELISA to assess translocation. Finally, the regulatory effects of AMR on the A20/TRAF6/NF-κB signaling pathway were validated using Western blotting. Spearman’s correlation analysis was employed to integrate microbiota changes with host inflammatory phenotypes.

**Results:**

AMR significantly ameliorated CDHFD-induced diarrhea. Mechanistically, AMR remodeled the gut microecology by enriching beneficial bacteria, particularly *Lachnospiraceae_NK4A136* and *norank_f_Muribaculaceae*. Concurrently, it increased the number of goblet cells and regulated the expression of tight junction proteins to repair intestinal barrier damage and reverse hyperpermeability. The restoration of barrier function effectively blocked the systemic translocation of LPS, which subsequently inhibited the hyperactivation of the NF-κB signaling pathway, thereby reducing systemic inflammation and ultimately alleviating diarrhea.

**Conclusion:**

AMR exerts protective effects against CDHFD-induced diarrhea through microbiota-driven intestinal barrier restoration, which sequentially blocks the activation of the LPS/NF-κB inflammatory pathway.

**Supplementary Information:**

The online version contains supplementary material available at 10.1186/s13020-026-01467-0.

## Highlights

1. AMR ameliorates diarrhea and systemic inflammation induced by a high-fat diet and cold drinks in mice.
2. AMR restores intestinal homeostasis by modulating gut microbiota and repairing the mucosal barrier.
3. AMR exerts an indirect pharmacological effect by preventing gut-derived LPS translocation, thereby suppressing downstream inflammatory pathways.

**Supplementary Information:**

The online version contains supplementary material available at 10.1186/s13020-026-01467-0.

## Introduction

The global incidence of chronic diarrhea is steadily rising, largely driven by shifts in modern dietary habits and lifestyles. Research indicates that excessive intake of high-fat and high-sugar foods may lead to chronic diarrhea, particularly in individuals with gastrointestinal disorders such as irritable bowel syndrome [1, 2]. Additionally, cold stimulation can impair digestive system function and exacerbate symptoms such as diarrhea and abdominal pain, especially in patients with irritable bowel syndrome, colitis, and pancreatic insufficiency [3–5]. Although the combination of cold beverages and high-fat foods has become a common dietary pattern in modern society, research on the potential risks associated with their co-ingestion remains limited.

Maintaining the integrity of the intestinal mucosal barrier is crucial for gastrointestinal homeostasis and immune defense. Disruption of this barrier increases paracellular permeability, allowing luminal endotoxins, particularly lipopolysaccharide (LPS), to translocate into the bloodstream [6]. This LPS translocation triggers a cascade of local and systemic inflammatory responses. Consequently, the release of inflammatory mediators further damages intestinal epithelial tight junctions, creating a vicious cycle that inhibits water absorption, accelerates intestinal peristalsis, and ultimately leads to persistent diarrhea [7]. Notably, our previous studies have demonstrated that the a cold drinks and high-fat diet (CDHFD) is a potent trigger for this exact pathological process. CDHFD severely impairs digestive function and directly drives this LPS-induced inflammatory cycle, thereby exacerbating gastrointestinal disorders [8].

AMR, a medicinal plant of the Asteraceae family, has been utilized for over two millennia and was first documented in the *Shennong Bencao Jing*. Historically, it has been widely used to treat conditions such as bloating, diarrhea, and edema. Modern pharmacological research corroborates its traditional applications, revealing that AMR possesses significant capabilities in regulating gastrointestinal motility, modulating gut microbiota, and mitigating intestinal inflammation [9, 10]. Owing to its well-established safety profile and historical efficacy, AMR is not only prescribed in contemporary clinical therapy but is also widely incorporated into functional health foods, demonstrating significant value in health promotion.

However, despite its extensive clinical and dietary application, the precise ameliorative effects and underlying molecular mechanisms of AMR against diarrhea induced specifically by modern unhealthy dietary patterns (such as CDHFD) remain largely unexplored. Therefore, this study aims to elucidate the therapeutic efficacy and mechanisms of AMR on CDHFD-induced diarrhea. By comprehensively evaluating behavioral performance, intestinal barrier integrity, systemic inflammation, gastrointestinal hormones, and gut microbiota, we seek to provide robust scientific evidence for the application of AMR as a novel nutritional and therapeutic intervention for diet-related gastrointestinal disorders.

## Materials and methods

### Materials and reagents

AMR was provided by Zhejiang International Pharmaceutical Co., Ltd. (Zhejiang, China) and was authenticated as the rhizome of *Atractylodes macrocephala Koidz.* by Professor Bo Zhu at Zhejiang Chinese Medical University. The high-fat diet (SY19004) was purchased from Shuyu Biotechnology Co., Ltd. (Shanghai, China). Beer was purchased from Tsingtao Brewery Co., Ltd. (Qingdao, China).

ELISA kits for measuring serum levels of lipopolysaccharide (LPS, MM-36870O1), interleukin-6 (IL-6, MM-0163M1), gastrin (GAS, MM-44405M2), tumor necrosis factor-alpha (TNF-α, MM-0132M1), vasoactive intestinal peptide (VIP, MM-0446M2), motilin (MTL, MM-0492M2), 5-hydroxytryptamine (5-HT, MM-0443M2), d-lactic acid (d-LA, MM-0336M1), interleukin-1 (IL-1, MM-0039M1), and diamine oxidase (DAO, MM-0228M1) were acquired from Jiangsu Meimian Industrial Co., Ltd. (Jiangsu, China). Alcian Blue-PAS (AB-PAS, CR2203206) and hematoxylin and eosin (H&E) staining kits were provided by Nanjing Jiancheng Bioengineering Institute (Nanjing, China).

Primary antibodies against TRAF6 (ab40675) and β-actin (ab8226) were purchased from Abcam (Cambridge, UK). Antibodies targeting IκBα (4812), NF-κB p65 (3033), p-IκBα (2859), and p-NF-κB p65 (8242) were acquired from Cell Signaling Technology (MA, USA). Antibodies against A20 (5630), Claudin-1 (28674-1-AP), Occludin (27260-1-AP), Claudin-2 (26912-1-AP), ZO-1 (21773-1-AP), and a secondary antibody IgG (H+L) (SA00001-2) were sourced from Proteintech Group (Wuhan, China). DAPI (AR1177) and anti-fluorescence quenching reagent were supplied by Boster Biological Technology (CA, USA).

### Preparation of AMR aqueous extract

Briefly, 1500 g of AMR was soaked in a 12-fold volume of water (v/w) for 30 min. The mixture was then extracted using a decoction machine at 100 °C for 1 h, followed by filtration. The remaining medicinal residue was subjected to the same extraction process two additional times. Subsequently, all filtrates were combined and concentrated to 0.75 g/mL (equivalent to the crude drug) using a rotary evaporator at 60 °C. The final concentrated extract was stored at − 20 °C for subsequent experiments.

### Examination of AMR’s chemical components

UPLC-ESI-MS (Waters, Milford, MA, USA) was used to qualitatively analyze the chemical components of AMR. Following the extraction of the AMR decoction twice with n-butanol, the solvent was removed through rotary evaporation. The dried extract was then redissolved in methanol. After thorough vortexing, the mixture was centrifuged at 12,000×*g* for 5 min. The resulting supernatant was collected and filtered through a 0.22 μm membrane for further analysis.

Chromatographic separation was performed on an ACQUITY UPLC BEH C18 column. The column temperature was maintained at 30 °C, with a flow rate set at 0.2 mL/min and an injection volume of 4 μL. Mobile phase A consisted of 0.5% formic acid in water (v/v), while mobile phase B was acetonitrile. A linear gradient elution was applied as follows: 0–8.0 min, 10%-48% B; 8.0–12.0 min, 48%-50% B; 12.0–16.0 min, 50%-70% B; 16.0–18.0 min, 70%-80% B; and 18.0–26.0 min, 80% B [11].

Mass spectrometry was conducted using a ZenoTOF 7600 system equipped with an electrospray ionization (ESI) source. The operating temperature was set at 550 °C. The pressures for ion source gases 1 and 2 were maintained at 55 psi, and the curtain gas at 35 psi. An ion spray voltage of 5500 V was applied, alongside a collision energy of 35 V and a collision energy spread (CES) of 15 V. To identify the composition of AMR, the acquired data were processed using PeakView software and compared against the MassBank database.

High-performance liquid chromatography (HPLC) was employed for the quantitative analysis of atractylenolide I, II, and III in the AMR solution (0.1 g/mL). Separation was achieved on an InertSustain C18 column maintained at 25 °C. The flow rate was established at 1.0 mL/min, the injection volume at 20 μL, and the detection wavelength fixed at 220 nm. The mobile phase comprised 0.1% aqueous formic acid (A) and acetonitrile (B). The gradient program was configured as follows: 0–20 min, 50%–60% A; 20–30 min, 60%–70% A; 30–35 min, 70%–100% A; 35–40 min, 100% A; and 40–45 min, 100%–50% A.

The total polysaccharide content was evaluated using the phenol–sulfuric acid method. To construct the standard curve, varying volumes (0.3 to 1.2 mL in 0.1 mL increments) of a 1 mg/mL glucose standard solution were added to test tubes, and distilled water was added to reach a total volume of 2 mL. Subsequently, 1 mL of 5% phenol solution and 5 mL of concentrated sulfuric acid were sequentially added. The mixture was incubated for 20 min in a boiling water bath and then rapidly cooled to room temperature using an ice-water bath. The absorbance was measured at 490 nm. Finally, the AMR solution was processed following the same procedure, and its total polysaccharide content was calculated based on the established standard curve.

### Animals and treatment

Forty male ICR mice (21 ± 2 g) were obtained from the Animal Supply Center of the Zhejiang Academy of Medical Sciences. Male mice were exclusively used to eliminate potential confounding effects of the female estrous cycle on metabolic and inflammatory responses, as well as gut microbiota composition. Animals were housed in a Specific Pathogen-Free (SPF) facility at Zhejiang Chinese Medical University under standard conditions (22 ± 2 °C, 55 ± 10% humidity, 12-h light/dark cycle).

**Fig. 1:**
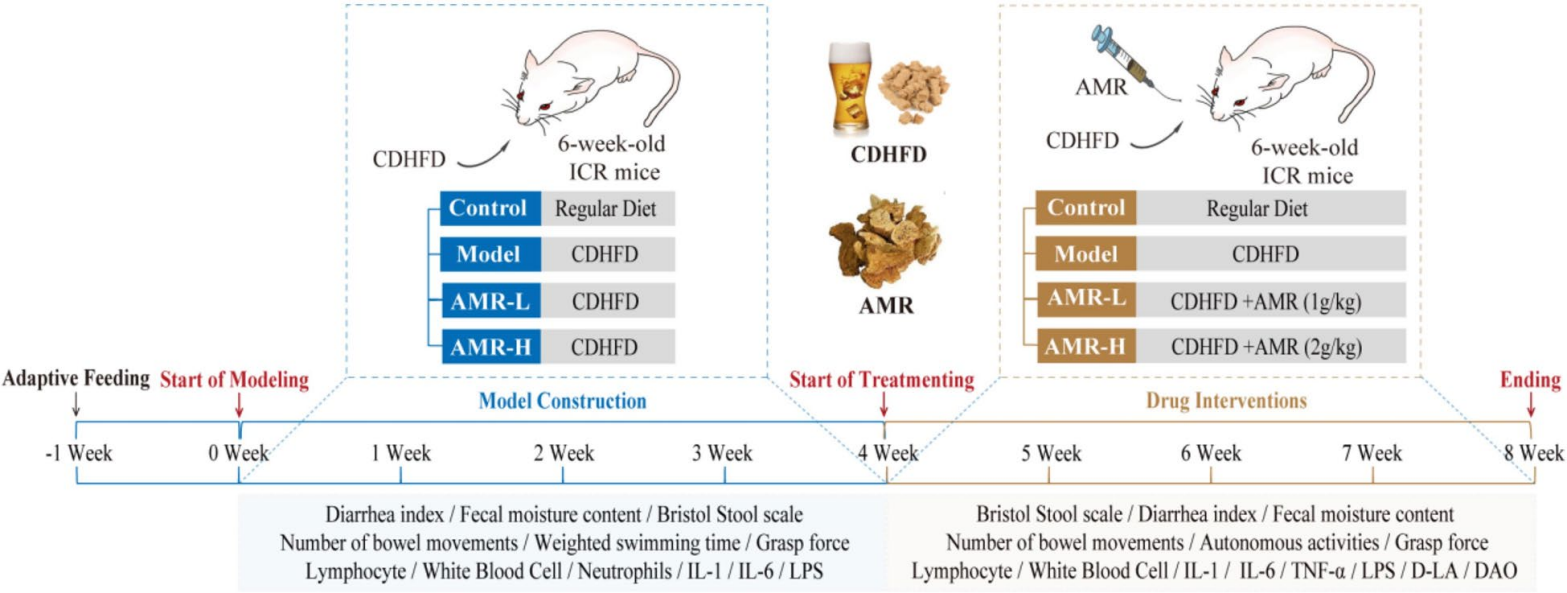
Experimentation protocols

Following a one-week acclimatization, the mice were randomly assigned to four groups (n = 10 per group): Control, Model, AMR-L (1 g/kg/day), and AMR-H (2 g/kg/day) (Fig. 1). To induce intestinal stress and diarrhea, mice (except for the control group) were subjected to a combined unhealthy dietary pattern, abbreviated as CDHFD. The CDHFD model consisted of a high-fat, high-cholesterol diet (15% pork fat, 0.8% cholesterol) combined with the administration of cold beer (4 °C, 4% vol). The cold beer was administered at 10 mL/kg/day for the first two weeks, and increased to 20 mL/kg/day for weeks three and four.

Upon successful model establishment at the end of week 4, corresponding drug interventions were initiated from the fifth week onward. The administered doses of AMR were determined by converting the clinical human dosage (6–12 g/day) recommended by the Chinese Pharmacopoeia using the body surface area (BSA) normalization method. To simulate persistent unhealthy dietary habits observed in clinical settings, the CDHFD conditions were maintained throughout the medication period.

At the end of the experiment, all mice were euthanized. Blood, intestinal tissues, and feces were collected and immediately stored at − 80 °C for subsequent analyses. All animal experiments were approved by the Animal Ethics Committee of Zhejiang Chinese Medical University (Approval No. 20240108-08) and conducted in strict adherence to the ARRIVE guidelines, the U.K. Animals (Scientific Procedures) Act 1986, and the EU Directive 2010/63/EU.

### Defecation test and feacal characteristics

#### Bristol stool scale

**Fig. 2:**
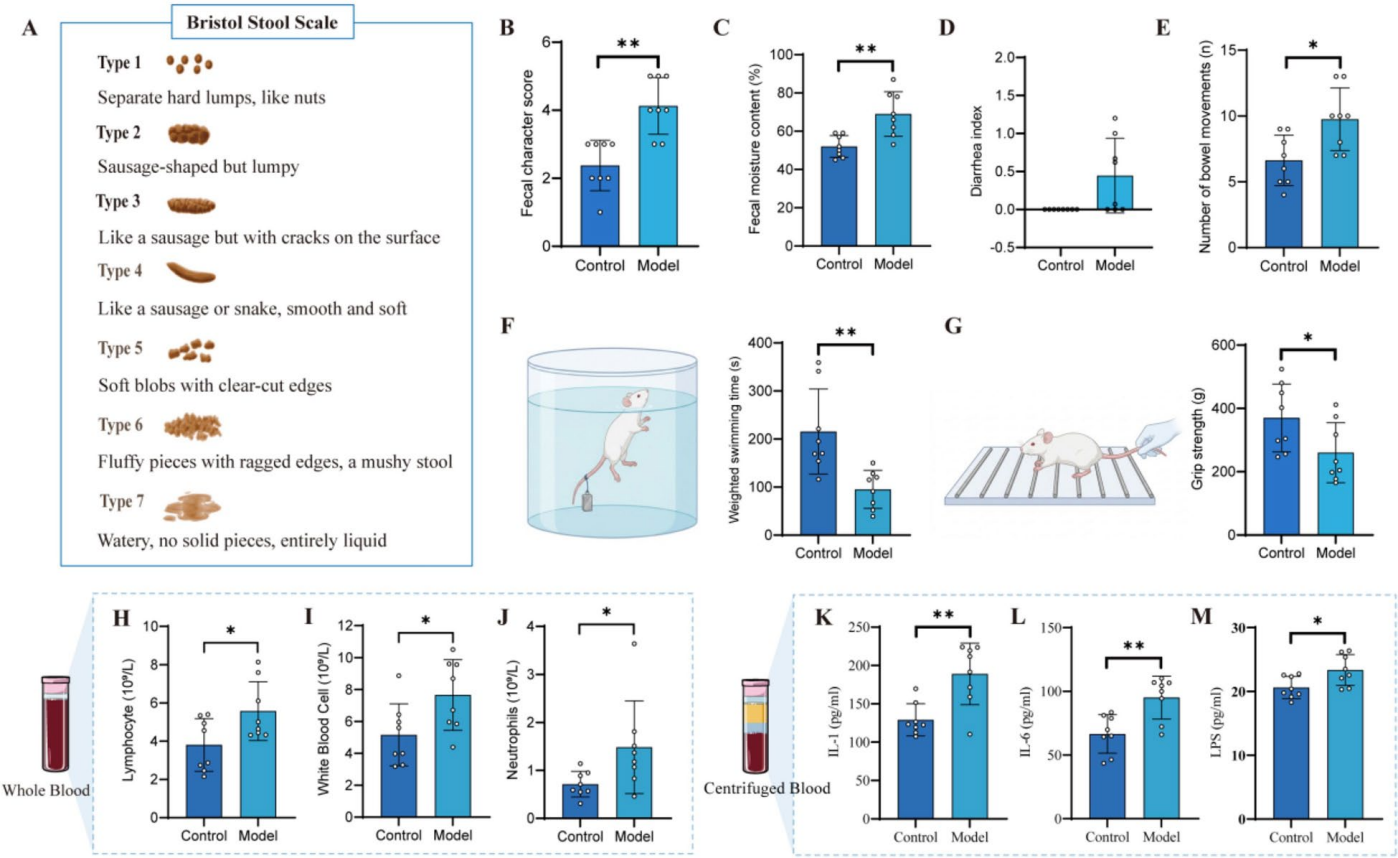
CDHFD-induced diarrhea with fatigue and systemic inflammation. **A** Bristol stool scale. **B** Fecal character score. **C** Fecal moisture content. **D** Diarrhea index. **E** Number of bowel movements. **F** Weighted swimming time. **G** Grip strength. **H**–**J** Counts of lymphocytes, white blood cells, and neutrophils in the blood. **K**–**M** Concentrations of IL-1, IL-6, and LPS in the serum. Data are presented as mean ± SD (n = 8 per group). Statistical significance was evaluated using Student’s *t*-test or the Mann–Whitney *U* test. * *P* < 0.05, ** *P* < 0.01

Fecal characteristics were evaluated using an adapted Bristol stool scale (Fig. 2A). The scoring criteria were defined as follows: Score 1, separate hard and dense lumps; Score 2, sausage-shaped but lumpy stools; Score 3, sausage-shaped stools with surface fissures; Score 4, smooth and snake-like stools; Score 5, soft blobs with smooth edges that are easy to pass; Score 6, loose and mushy stools with rough edges; and Score 7, watery or pasty feces indicative of severe diarrhea [12].

#### Faecal water content rate

Fresh fecal pellets from each mouse were collected and immediately weighed to record their initial wet weight. Subsequently, the samples were dried in an oven at 60 °C for 12 h and re-weighed to obtain their dry weight. The fecal water content was calculated using the following formula: [(wet weight − dry weight)/wet weight] × 100%.

#### Defecation frequency and diarrhea index

To evaluate the defecation frequency and diarrhea index, mice were individually placed in cages lined with filter paper for a continuous 4-h observation period. During this time, the filter paper was replaced every 30 min to accurately record the defecation events and prevent the overlapping of fecal stains. The severity of diarrhea was graded into four levels based on the diameter of the fecal stain left on the filter paper: Grade 1, < 1 cm; Grade 2, 1–2 cm; Grade 3, 2–3 cm; and Grade 4, > 3 cm [13, 14].

Based on these observations, the relevant parameters were calculated using the following formulas:

1. Diarrhea rate = (Number of loose stools/Total number of stools) × 100%;
2. Average diarrhea grade = Sum of the grades of all loose stools/Number of loose stools;
3. Diarrhea index = Diarrhea rate × Average diarrhea grade.

### Behavioural tests

#### Weight-loaded forced swimming test

A weight-loaded forced swimming test was performed to evaluate physical endurance. Briefly, a lead weight equivalent to 10% of each mouse’s body weight was attached to the base of its tail. The mice were then individually placed into a water tank (water depth: 30 cm; temperature: 25 ± 1 °C). The time from being placed in the water until complete exhaustion was recorded as the exhaustive swimming time. Exhaustion was defined as the point at which the mouse failed to return to the water surface to breathe within a 10-s period [15].

#### Autonomous activity test

To assess the autonomous activity of the mice, a multipurpose mouse autonomous activity recorder was utilized. The mice were initially placed in the testing apparatus and allowed to acclimate to the environment for 5 min. Following this acclimatization period, the number of movements for each mouse was continuously recorded over the subsequent 5 min.

#### Grip strength test

The mice were placed on a grip strength meter, and the maximum force they exerted while grasping the device was recorded. This test was repeated three times for each mouse, and the average value was calculated as the final result.

### Gut microbiota analysis

Total genomic DNA was extracted from the intestinal samples using the E.Z.N.A.® DNA kit, and its concentration was subsequently measured. The V3–V4 hypervariable region of the bacterial 16S rRNA gene was amplified utilizing the primer pair 338F/806R. The PCR products were evaluated by 2% agarose gel electrophoresis, followed by purification with the AxyPrep DNA Gel Purification Kit and quantification via a Quantus™ Fluorometer. Finally, the purified amplicons were subjected to paired-end sequencing on an Illumina MiSeq PE300 platform.

All data analyses were performed on the Majorbio Cloud Platform (https://cloud.majorbio.com). Specifically, alpha diversity indices, including Chao and Shannon, were calculated, and the Wilcoxon rank-sum test was used to compare alpha diversity between groups. Principal component analysis (PCA) and partial least squares discriminant analysis (PLS-DA) were performed to assess the similarity of microbial community structures among samples. Analysis of similarities (ANOSIM) was utilized to calculate *P*-values and corresponding R-statistics to determine the statistical significance of differences in microbial community structure between groups. LEfSe analysis was conducted with a logarithmic LDA score threshold of 2.0 and a *P* < 0.05 to identify bacterial taxa with significantly different abundances between groups, from the phylum to the genus level. Linear regression analysis was used to evaluate the influence of major physiological indices, identified by db-RDA, on microbial alpha and beta diversity indices.

### Blood routine test

Blood samples were analyzed using an automated hematology analyzer (XP-100; Sysmex, Japan) to quantify white blood cell and lymphocyte counts.

### ELISA

The concentrations of GAS, MTL, VIP, TNF-α, LPS, D-LA, IL-1, 5-HT, IL-6, and DAO in the samples were measured using specific ELISA kits according to the manufacturer’s instructions.

### Western blot analysis

The expression levels of TRAF6, A20, IκB, p65, p-IκB, p-p65, and β-actin proteins in colon tissues were assessed by Western blotting. Briefly, total protein was extracted from tissues samples using RIPA lysis buffer supplemented with protease and phosphatase inhibitors. Protein concentrations were determined using a NanoDrop One spectrophotometer (Thermo Fisher Scientific, USA). The proteins were then separated by 8% or 12% SDS-PAGE and transferred onto nitrocellulose membranes. After blocking with 5% BSA for 1 h at room temperature, the membranes were incubated overnight at 4 ℃ with primary antibodies against TRAF6 (1:5000), A20 (1:1000), IκB (1:1000), p65 (1:1000), p-IκB (1:1000), p-p65 (1:1000), and β-actin (1:10,000). Subsequently, the membranes were incubated with HRP-conjugated secondary antibodies (1:5000) for two hours at room temperature. Protein bands were visualized using an ECL detection system, and the band intensities were quantified using ImageJ software (NIH, USA).

### H&E and AB-PAS staining

Colon tissues samples were first fixed in 4% paraformaldehyde, dehydrated through a graded ethanol series, cleared in xylene, and embedded in paraffin. The paraffin blocks were then cut into 4-μm-thick sections using a rotary microtome. Subsequently, the sections were deparaffinized, rehydrated, and subjected to Hematoxylin and Eosin (H&E) staining and Alcian Blue-Periodic Acid-Schiff (AB-PAS) staining. H&E staining was performed to evaluate the basic histopathological morphological changes. AB-PAS staining was conducted to identify and differentiate mucus types, with acidic mucins appearing blue and neutral mucins appearing purple-red. Finally, the stained sections were observed and imaged using a light microscope (Olympus BX53, Olympus, Japan).

### Immunofluorescence (IF) staining

Paraffin-embedded ileum and colon tissues sections (4 μm) were deparaffinized in xylene and rehydrated through a graded ethanol series. Antigen retrieval was performed by heating the sections in EDTA buffer (pH 9.0). After washing with PBS, the tissues were blocked with 3% BSA for 1 h at room temperature to prevent non-specific binding. The sections were then incubated overnight at 4 ℃ with primary antibodies against occludin (1:500), claudin-1 (1:1000), ZO-1 (1:500), and claudin-2 (1:1000). Following three washes in PBS, the sections were incubated with fluorophore-conjugated secondary antibodies for 60 min at room temperature in the dark. Nuclei were counterstained with DAPI, and the sections were mounted with an anti-fade mounting medium. Finally, images were acquired using a fluorescence microscope (Axio Scope.A1, Carl Zeiss, Germany).

### Statistical analysis

Statistical analyses were performed using IBM SPSS Statistics version 31.0 (IBM Corp., Armonk, NY, USA). All data are presented as the mean ± standard deviation (SD). Normality of the data (n = 6–8 per group) was evaluated using the Shapiro–Wilk test. For comparisons between two independent groups, the unpaired Student’s *t*-test was used for normally distributed data, while the Mann–Whitney *U* test was employed for non-normally distributed data. For comparisons among three or more groups, normally distributed data were analyzed using a one-way analysis of variance (ANOVA). Prior to post hoc analysis, the homogeneity of variances was assessed using Levene’s test. The Least Significant Difference (LSD) test was applied for multiple comparisons if variances were equal, whereas Dunnett’s T3 test was used if variances were unequal. Non-normally distributed multi-group data were analyzed using the Kruskal–Wallis test. Statistical significance was set at *P* < 0.05.

## Results

### CDHFD-induced diarrhea with fatigue and induces inflammation in mice

Dietary patterns are closely related to intestinal health, and defecation habits and faecal traits can reflect intestinal health [16, 17]. To evaluate the effects of the CDHFD on intestinal function and physical status, we monitored fecal traits and physical endurance in mice. Compared with the control group, mice in the CDHFD model group exhibited severe diarrheal symptoms, evidenced by significantly elevated Bristol stool scores (Fig. 2A, B; *P* < 0.01), fecal water content (Fig. 2C; *P* < 0.01), diarrhea index (Fig. 2D; *P* < 0.01), and increased defecation frequency (Fig. 2E; *P* < 0.05). Furthermore, the CDHFD-fed mice displayed obvious signs of physical fatigue. This was demonstrated by a marked reduction in exhaustive weight-bearing swimming time (Fig. 2F; *P* < 0.01) and diminished grip strength (Fig. 2G; *P* < 0.05) compared to the controls.

Research has shown that unhealthy diets, such as excessive intake of high-calorie, high-fat, and sugar-rich foods, can cause inflammation and trigger various chronic diseases [18]. Additionally, our earlier research has demonstrated that CDHFD can result in inflammatory responses [8]. Given that unhealthy diets are closely associated with inflammatory responses, we next evaluated the hematological and inflammatory profiles of the mice. Complete blood count analysis revealed a significant elevation in the circulating levels of lymphocytes (Fig. 2H; *P* < 0.05), leukocytes (Fig. 2I; *P* < 0.05), and neutrophils (Fig. 2J; *P* < 0.05) in the model group. Consistently, serum concentrations of pro-inflammatory cytokines, including IL-1 (Fig. 2K; *P* < 0.01) and IL-6 (Fig. 2L; *P* < 0.01), as well as the endotoxin LPS (Fig. 2M; *P* < 0.05), were markedly upregulated. Collectively, these findings indicate that the CDHFD successfully induced diarrhea, fatigue, and a state of systemic inflammation in mice.

### Analysis of the composition of AMR

**Fig. 3:**
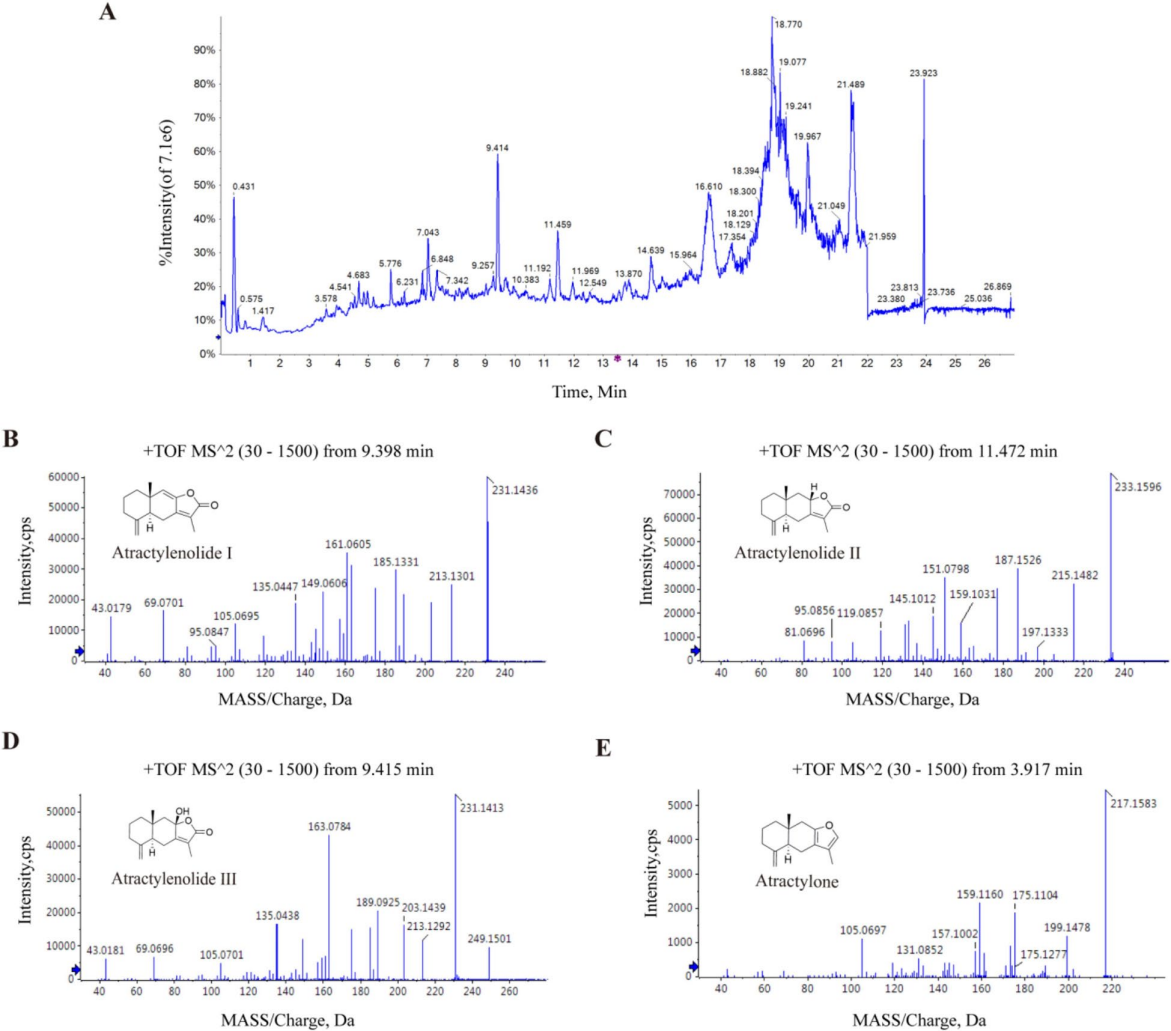
Identification of the main chemical constituents of AMR. **A** Total ion chromatogram of AMR obtained by UPLC-ESI–MS. **B**–**E** MS/MS spectra of Atractylenolide I, Atractylenolide II, Atractylenolide III, and Atractylone, respectively

In this study, AMR extracted from the n-butanol fraction was analyzed using UPLC-ESI-MS. As shown in Fig. 3A and Table 1, a total of 21 compounds were identified, including Atractylenolide I (Fig. 3B), Atractylenolide II (Fig. 3C), Atractylenolide III (Fig. 3D), and Atractylone (Fig. 3E), among others. Furthermore, the concentrations of Atractylenolide II, Atractylenolide III, and total polysaccharides in the AMR solution were determined to be 3.26 mg/mL, 16.81 mg/mL, and 147.4529 mg/mL, respectively.

**Table 1:** Identification of compounds in AMR samples by UPLC-EIS-MS

No	t_R/_min	Chemical constituents	Formula	[M+H]^+^		Error	m/z
				Calculated value	Measured Value		
1	0.443	(E)-deca-2-ene-4, -diyne-1,10-diol-1-*O*-β-d-apiofuranosyl-(1–6)-β-d-glu-copyranoside	C_21_H_29_O_11_	457.1710	458.1846	1.8	133.0583, 116.0710, 70.0591
2	0.440	(1’’R,2″S,3″R)-1’’-hydroxylcrotonine	C_12_H_15_N_2_O_5_	267.0981	268.1034	− 7.3	158.1163, 136.0621
3	0.518	7-hydroxycoumarin	C_9_H_6_O_3_	162.0317	163.0386	− 1.9	145.0064, 135.0456, 85.0275, 45.0327
4	4.505	8 p-ethoxy atractylodes lactone IH	C_17_H_23_O_2_	259.1698	260.1614	− 1.9	214.1799, 158.1164
5	5.500	2-(3,7-dimethylocta-2,6-dien-1-y)1–6-methylcyclohexa-2,5-diene-1,4-dione	C_17_H_22_O_2_	259.1693	259.1684	− 1.3	259.1684, 223.0502,166.9772,123.1159
6	5.904	Scopoletin-7-*O*-β-d-xylopyranosyl-(1 → 6)-β-d)-glucopyranoside	C_21_H_27_O_13_	487.1452	488.1480	− 1.9	359.1047
7	7.062	P-Hydroxyphenyl ethyl phenylacetate	C_16_H_16_O_3_	256.1099	257.1200	− 2.3	239.1054,197.0968,173.0968, 153.0688,129.0687
8	7.362	Atractylenolide V	C_15_H_20_O_4_	265.1434	265.1479	2.6	247.1324, 219.1361, 147.1170
9	7.710	Vessane dienone	C_15_H_22_O	218.1671	219.1736	− 2.0	201. 1639, 177.1279, 161.1329, 105.0688
10	9.414	Eucalyptus isospoonet	C_15_H_24_O	220.1827	221.1889	− 2.1	221.1889, 203. 1790, 175.1478
11	9.415	Atractylenolide III	C_15_H_20_O_3_	249.1485	249.1501	1.9	249.1501, 231.1413, 163.0784,135.0438
12	10.234	Diacetamidophorinol	C_17_H_20_O_4_	289.1434	289.1437	0	289.1437
13	10.356	8β-ethoxyasterolid	C_17_H_24_O_3_	276.1725	277.1790	− 2.6	235.1717,217.1590,199.1477,157.1009, 105.0703
14	11.472	Atractylenolidel (isomer)	C_15_H_20_O_2_	232.1463	233.1596	− 0.9	215.1482,187.1526,151.0798
15	11.472	Atractylenolidell	C_15_H_20_O_2_	232.1463	233.1596	− 1.2	215. 1482,187.1526, 159.1031,151.0798
16	13.0660	8β-methoxy atracolactone I	C_16_H_22_O_3_	262.1569	263.1630	0.6	231.1381、203.1787,163.0733
17	15.141	Atractylol	C_15_H_16_O	222.1984	223.1044	− 7.0	207.0310,149.0233,131.0008
18	15.55	Atractylone	C_15_H_20_O	216.1514	217.1583	− 2.1	199. 1478, 175.1104, 159.1160, 105.0697
19	16.3800	Atractylenolide I	C_15_H_18_O_2_	230.1307	231.1436	− 0.9	213. 1301, 185. 1331, 161.0605,135.0447,105.0695
20	17.278	Atractyloside B	C_21_H_38_O_10_	450.2465	451.2499	− 3.5	433.2412, 187.0593
21	19.631	Biatractylolide	C_30_H_38_O_4_	463.2843	463.2804	− 1.2	463.2804, 231.1379

### AMR ameliorates CDHFD-induced diarrhea and fatigue

**Fig. 4:**
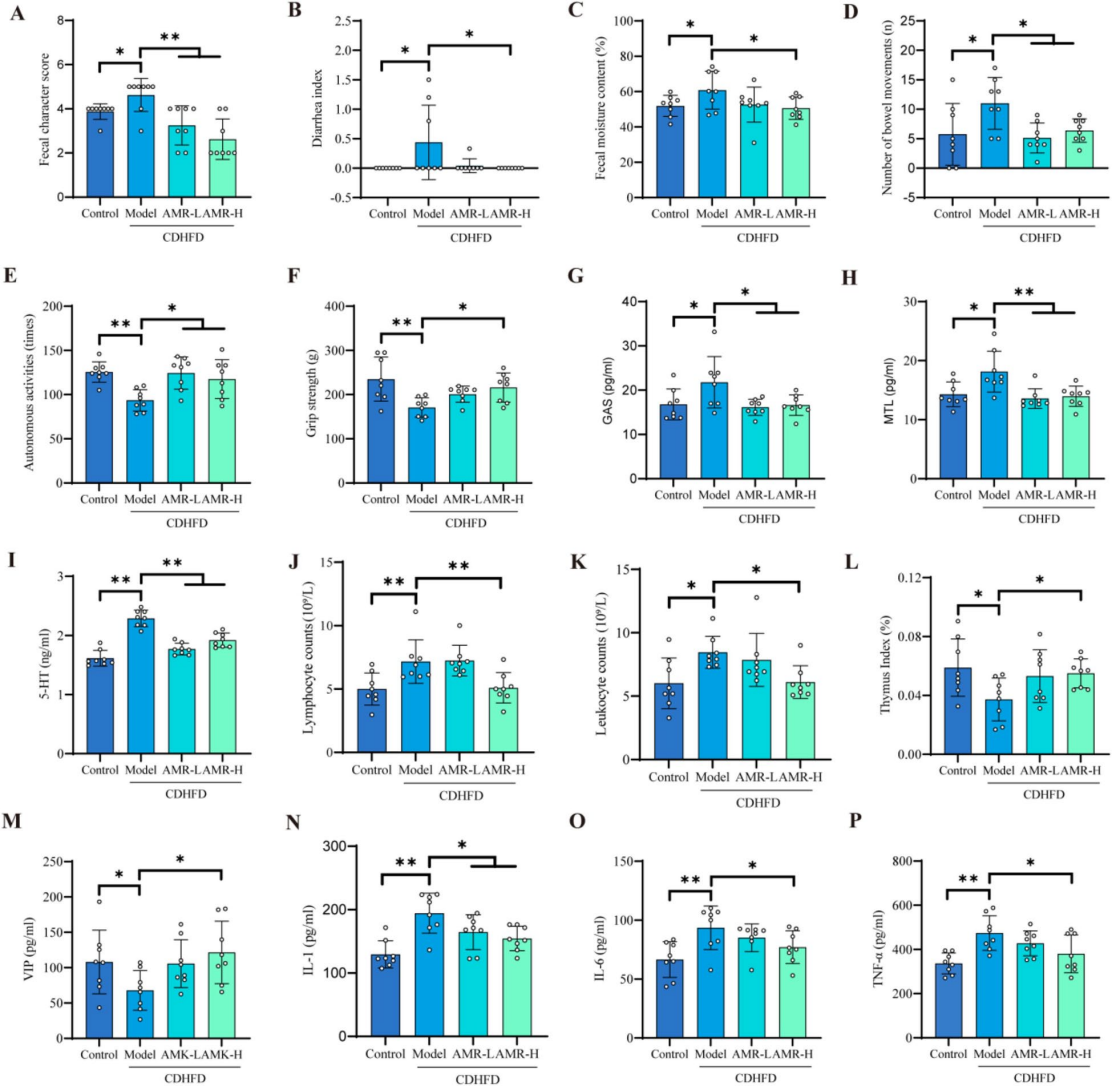
Ameliorative effects of AMR on CDHFD-induced diarrhea, fatigue, abnormal gastrointestinal hormone secretion, and systemic inflammation. **A** Bristol stool score. **B** Diarrhea index. **C** Fecal water content. **D** Number of bowel movements. **E** Autonomous activities. **F** Grip strength. **G**–**I** Serum levels of GAS, MTL, and 5-HT. **J**, **K** Blood lymphocyte and leukocyte counts. **L** Thymus index. **M**–**P** Serum concentrations of VIP, IL-1, IL-6, TNF-α. Data are presented as mean ± SD, n = 8. Statistical significance was evaluated using one-way ANOVA (followed by LSD or Dunnett’s T3 post-hoc tests) or the Kruskal–Wallis test. * *P* < 0.05, ** *P* < 0.01

The present research investigated the impact of AMR on diarrhea induced by CDHFD, focusing on defecation patterns and characteristics of feces. The modeling with CDHFD led to diarrhea in mice, which was indicated by significant increases in the average Bristol stool score (from 3.38 to 4.63, *P* < 0.05), the diarrhea index (from 0.00 to 0.44, *P* < 0.05), fecal water content (from 51.90 to 60.81%, P < 0.05), and defecation frequency (from 5.75 to 11.00, *P* < 0.05) (Fig. 4A–D). Following treatment, the Bristol stool scores in the AMR-L and AMR-H groups fell to 3.25 and 2.63, respectively (Fig. 4A; *P* < 0.01). Additionally, the number of bowel movements was reduced to 5.13 and 6.38, respectively (Fig. 4D; *P* < 0.05). Notably, when 2 g/kg of AMR was administered, the mice’s diarrhea index dropped to 0.00 (Fig. 4B; *P* < 0.05), and there was a reduction to 50.62% in the fecal water content (Fig. 4C; *P* < 0.05). These findings imply that AMR can greatly alleviate diarrhea caused by CDHFD in mice.

We assessed the effect of AMR on the behavior of CDHFD mice using autonomous activities and grasping power. The results indicated that the number of autonomous activities (Fig. 4E; *P* < 0.01) and grasping power (Fig. 4F; *P* < 0.01) were markedly lower in the model group mice. Following treatment with AMR, the number of autonomous activities of mice was notably increased (Fig. 4E; *P* < 0.05), and the grasping power in the AMR-H group was markedly improved (Fig. 4F; *P* < 0.05). These results suggest that AMR can significantly ameliorate the fatigue and weakness of mice caused by CDHFD.

### AMR modulates CDHFD-induced abnormal gastrointestinal hormone secretion

Intestinal peristalsis is regulated by various gastrointestinal hormones that play a crucial role in the pathogenesis of diarrhea [19–22]. During diarrheal episodes, the serum levels of hormones such as GAS are significantly elevated, and this overproduction leads to intestinal hyperexcitability, thereby exacerbating diarrhea symptoms [23–25]. To evaluate the regulatory effect of AMR on gastrointestinal hormones in mice fed a CDHFD, we measured the serum concentrations of GAS, MTL, and 5-HT. The results demonstrated that the model group exhibited significantly higher serum concentrations of GAS (Fig. 4G; *P* < 0.05), MTL (Fig. 4H; *P* < 0.05), and 5-HT (Fig. 4I; *P* < 0.01). Furthermore, these elevated concentrations were significantly decreased following AMR intervention (Fig. 4G–I; *P* < *0.05 or P* < *0.01*). These findings indicate that AMR effectively regulates the secretion of hormones associated with intestinal motility.

### AMR reduces CDHFD-induced inflammation

Diet plays a crucial role in the onset and progression of inflammation [26]. To assess the effect of AMR on systemic inflammation in mice fed a CDHFD, blood lymphocyte counts, leukocyte counts, and the thymus index were examined. The results indicated that the model group exhibited significantly elevated counts of blood lymphocytes (Fig. 4J; *P* < 0.01) and leukocytes (Fig. 4K; *P* < 0.05). Following intervention, the AMR-H group showed a significant reduction in both lymphocyte and leukocyte counts (Fig. 4J, K; *P* < 0.01, 0.05). Furthermore, the thymus index, which was significantly decreased in the model group, was markedly restored in the AMR-H group (Fig. 4L; *P* < 0.05).

Pro-inflammatory cytokines, such as IL-1, IL-6, and TNF-α, serve as essential mediators in the inflammatory response. Conversely, VIP plays a crucial role in regulating inflammation by suppressing the expression of these pro-inflammatory factors [27]. We subsequently evaluated the serum concentrations of these markers. The findings revealed that VIP levels were significantly decreased in the model group but were markedly elevated following AMR-H treatment (Fig. 4M; *P* < 0.05). Correspondingly, the serum concentrations of IL-1 (Fig. 4N; *P* < 0.01), IL-6 (Fig. 4O; *P* < 0.01), and TNF-α (Fig. 4P; *P* < 0.01) were robustly increased in the model group. Both AMR-L and AMR-H treatments significantly reduced IL-1 levels (Fig. 4N; *P* < 0.05), while the AMR-H intervention also significantly decreased the levels of IL-6 (Fig. 4O; *P* < 0.05) and TNF-α (Fig. 4P; *P* < 0.05). Collectively, these cellular and cytokine profiles demonstrate that AMR effectively mitigates the systemic inflammation provoked by CDHFD.

### AMR restores the CDHFD-disrupted intestinal microbial barrier

Dietary habits profoundly shape the gut microbiota and influence the intestinal microecology [28–30]. To investigate the regulatory effect of AMR on gut dysbiosis, we evaluated the diversity, richness, overall community structure, and specific taxonomic composition of the gut microbiota, as well as their correlations with disease-related parameters.

#### AMR’s effects on the composition of the gut microbiota in CDHFD mice

**Fig. 5:**
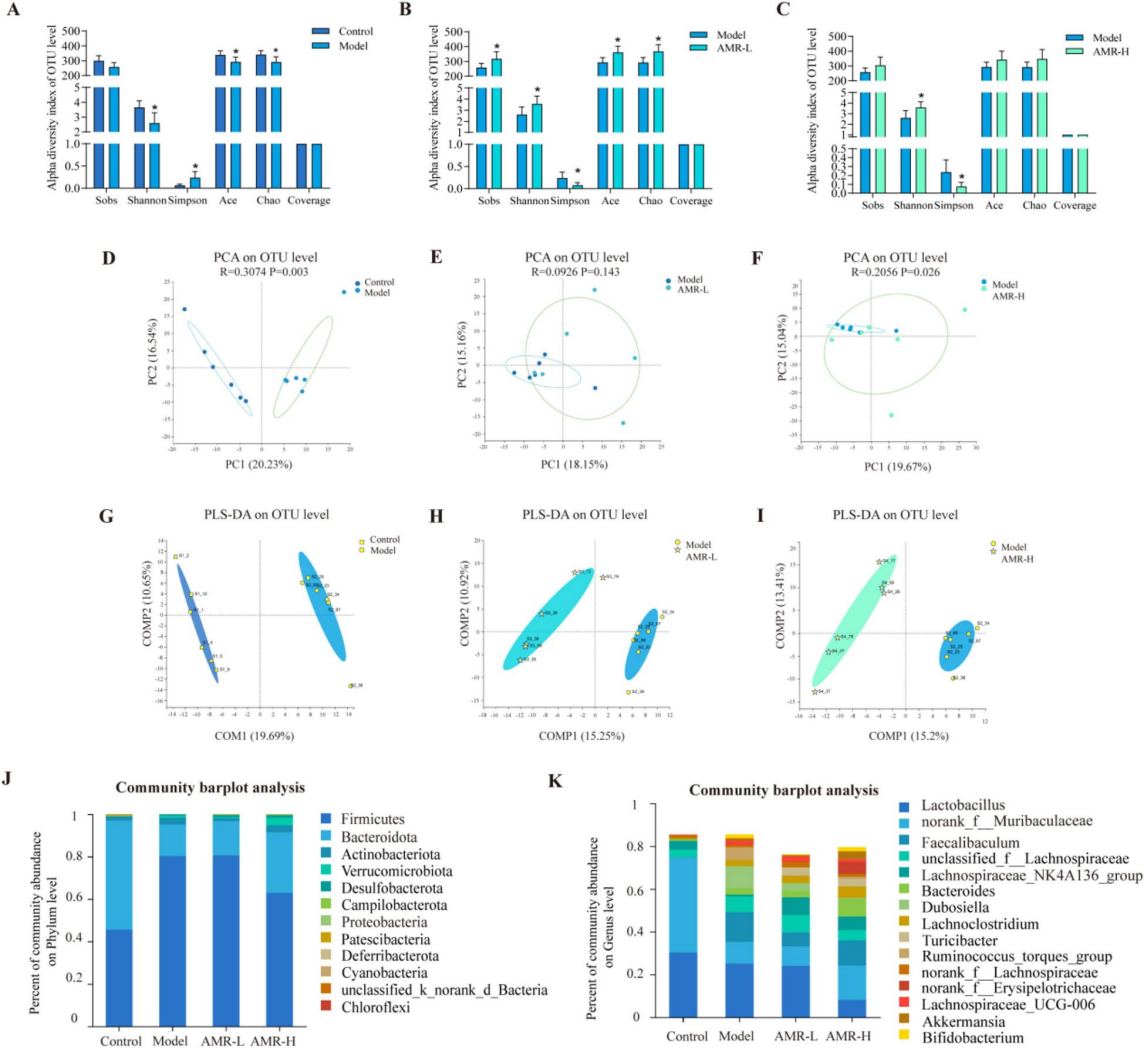
Effect of AMR on diversity and composition of the gut microbiota. **A**–**C** Alpha diversity analysis. **D**–**F** Principal component analysis (PCA) plots based on β-diversity. **G**–**I** Partial least squares discriminant analysis (PLS-DA) plots. The R-values and P-values presented in **D**–**I** were derived from the ANOSIM analysis. **J**, **K** Relative abundance of gut microbiota at the phylum and genus levels. Data are presented as mean ± SD, n = 6. Statistical significance was evaluated using Student’s t-test or the Mann–Whitney U test. * *P* < 0.05, ** *P* < 0.01

Through 16S rRNA sequencing of fresh fecal samples, a total of 2,151,705 raw sequences were obtained. After quality control, 1,880,579 valid sequences were generated. Alpha diversity analysis indicated that the model group exhibited a notably higher Simpson index while showing significantly lower values for the Shannon, Ace, and Chao indices compared to the control group (Fig. 5A; *P* < 0.05). Following intervention, the AMR-L group revealed significantly increased Sobs, Shannon, Ace, and Chao indices, along with a considerably reduced Simpson index (Fig. 5B; *c* < 0.05). Similarly, the AMR-H group showed a notable decrease in the Simpson index and a significant increase in the Shannon index (Fig. 5C; *P* < 0.05). These findings suggest that the CDHFD significantly reduces microbial community diversity and species richness in the mouse gut, whereas AMR intervention effectively reverses these deleterious effects.

The impact of AMR on the overall structure of gut microbial communities was assessed using β-diversity analysis via PCA and PLS-DA. The PCA score plot indicated a clear separation between the model and control groups (Fig. 5D; R = 0.3074; *P* < 0.01), implying a distinct shift in the intestinal microbiota structure. Compared with the model group, the AMR-L group did not show a statistically significant separation (Fig. 5E; R = 0.0926; *P* > 0.05); however, the AMR-H group exhibited a distinct structural separation (Fig. 5F; R = 0.2056; P < 0.05), indicating a profound modulation of the gut microbiota structure following high-dose AMR treatment. Consistently, PLS-DA revealed that samples from the model group were distinctly segregated from the control, shifting along the positive axis of COMP1 (Fig. 5G). In contrast, the AMR treatment groups were distinguishable from the model group, shifting back toward the negative direction of COMP1 (Fig. 5H, I). These results collectively suggest that CDHFD alters the configuration of the intestinal microbiota, which is partially restored toward a normal state by AMR intervention.

Taxonomic composition analysis was performed to identify specific microbial alterations. At the phylum level, Firmicutes and Bacteroidetes were the dominant phyla across all groups; although the dominant phyla remained consistent, their relative abundances varied notably among the groups (Fig. 5J). At the genus level, distinct variations in the composition and relative abundance of gut bacteria were observed (Fig. 5K). In the control group, the predominant genera included *Lactobacillus*, *norank_f_Muribaculaceae*, and *Lachnospiraceae_NK4A136_group*. Conversely, the key microorganisms enriched in the model group were *Lactobacillus*, *Faecalibaculum*, and *Dubosiella*. Following treatment, the primary genera in the AMR-L group were *Lactobacillus*, *norank_f_Muribaculaceae*, and *Lachnospiraceae_NK4A136_group*, while the AMR-H group consisted mainly of *Lactobacillus*, *Faecalibaculum*, and *norank_f_Muribaculaceae*.

#### Effect of AMR on specific gut microbiota taxa and their correlations with phenotypic parameters

**Fig. 6:**
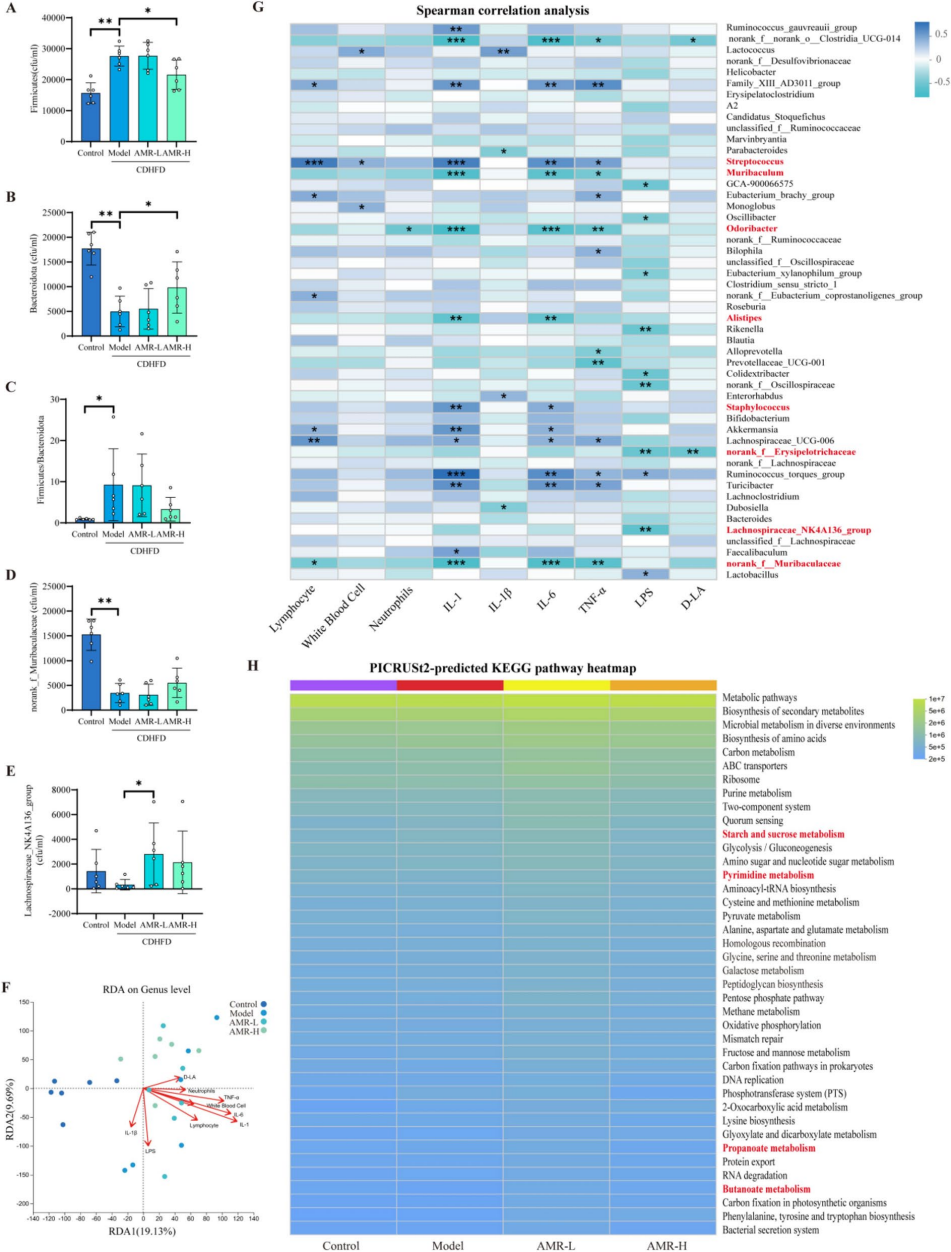
Effect of AMR on specific gut microbiota taxa and their correlations with disease-related parameters. **A**, **B** Relative abundance of Firmicutes and Bacteroidota. **C** The Firmicutes/Bacteroidota (F/B) ratio. **D**, **E** Relative abundance of *norank_f_Muribaculaceae* and *Lachnospiraceae_NK4A136_group*. **F** Redundancy analysis (RDA) of the gut microbiota and disease-related parameters. **G** Spearman correlation heatmap between specific microbial taxa and disease-related parameters. **H** Heatmap of KEGG pathways predicted by PICRUSt2. Data are presented as mean ± SD, n = 6. Statistical significance was evaluated using one-way ANOVA (followed by LSD or Dunnett’s T3 post-hoc tests) or the Kruskal–Wallis test. * *P* < 0.05, ** *P* < 0.01

At the phylum level, the most significant changes in relative abundance were observed in Firmicutes and Bacteroidota. The model group showed a significant increase in the abundance of Firmicutes (Fig. 6A; *P* < 0.01) and a sharp decrease in Bacteroidota (Fig. 6B; *P* < 0.01). Following the intervention, treatment with high-dose AMR (AMR-H) significantly reversed these trends, decreasing Firmicutes and increasing Bacteroidota (Fig. 6A, B; *P* < 0.05). Consequently, the Firmicutes/Bacteroidota (F/B) ratio, which was significantly elevated in the model group (Fig. 6C; *P* < 0.05), decreased after AMR-H intervention, although this reduction was not statistically significant (*P* > 0.05).

Research indicates that in various intestinal disorders, the abundance of *norank_f_Suribaculaceae* and *Lachnospiraceae_NK4A136_group* is typically significantly reduced [31, 32]. Consistent with this, our results revealed that the abundance of *norank_f_Muribaculaceae* was significantly depleted in the model group (Fig. 6D; *P* < 0.01), but was subsequently restored after AMR-H intervention. Furthermore, the abundance of *Lachnospiraceae_NK4A136_group*, which had also decreased in the model group, was significantly enriched following AMR-L intervention (Fig. 6E; *P* < 0.05). These results suggest that the CDHFD-induced dysbiosis of multiple specific bacterial taxa could be reversed to varying degrees by AMR treatment.

The relationships between the gut microbiota and disease-related parameters were further examined. RDA results revealed a substantial correlation between microbial taxa, such as *Lachnospiraceae_NK4A136_group*, and inflammatory parameters including IL-1, LPS, and IL-6 (Fig. 6F). Spearman correlation Spearman correlation analysis (Fig. 6G; *P* < *0.05 or P* < *0.01*) provided further details. Several taxa exhibited significant negative correlations with inflammatory markers: *norank_f_Muribaculaceae* negatively correlated with IL-1, TNF-α, IL-6, and lymphocytes; *Lachnospiraceae_NK4A136_group* with LPS; *norank_f_Erysipelotrichaceae* with D-LA and LPS; *Alistipes* with IL-6 and IL-1; and *Odoribacter* with IL-6, TNF-α, IL-1, and neutrophils. Conversely, *Staphylococcus* and *Muribaculum* were positively correlated with pro-inflammatory cytokines (IL-6, IL-1, and/or TNF-α). Notably, *Streptococcus* showed a broad positive correlation with IL-1, TNF-α, IL-6, leukocytes and lymphocytes. These findings indicate a strong association between alterations in the gut microbiota and host inflammatory responses.

Finally, PICRUSt2 functional prediction based on the KEGG database was performed to assess the metabolic potential of the intestinal microbiota (Fig. 6H). We found that pathways related to “butanoate metabolism” and “propanoate metabolism” were highly enriched in the direct synthesis of SCFAs, while “pyruvate metabolism” and “starch and sucrose metabolism” were highly enriched in the SCFA precursor and carbohydrate fermentation pathways.

### Effect of AMR on CDHFD-induced intestinal mechanical barrier damage

When the intestinal barrier is compromised, intestinal permeability increases, allowing harmful gut-derived macromolecules (such as endotoxins like LPS) to enter the bloodstream. This event not only triggers systemic immune activation but is also a critical driver of various diseases [33]. To evaluate the effects of AMR on the intestinal mechanical barrier, we examined histopathological changes in the intestinal tissues, measured serum concentrations of D-LA, LPS, and DAO, and assessed the expression levels of tight junction proteins.

**Fig. 7:**
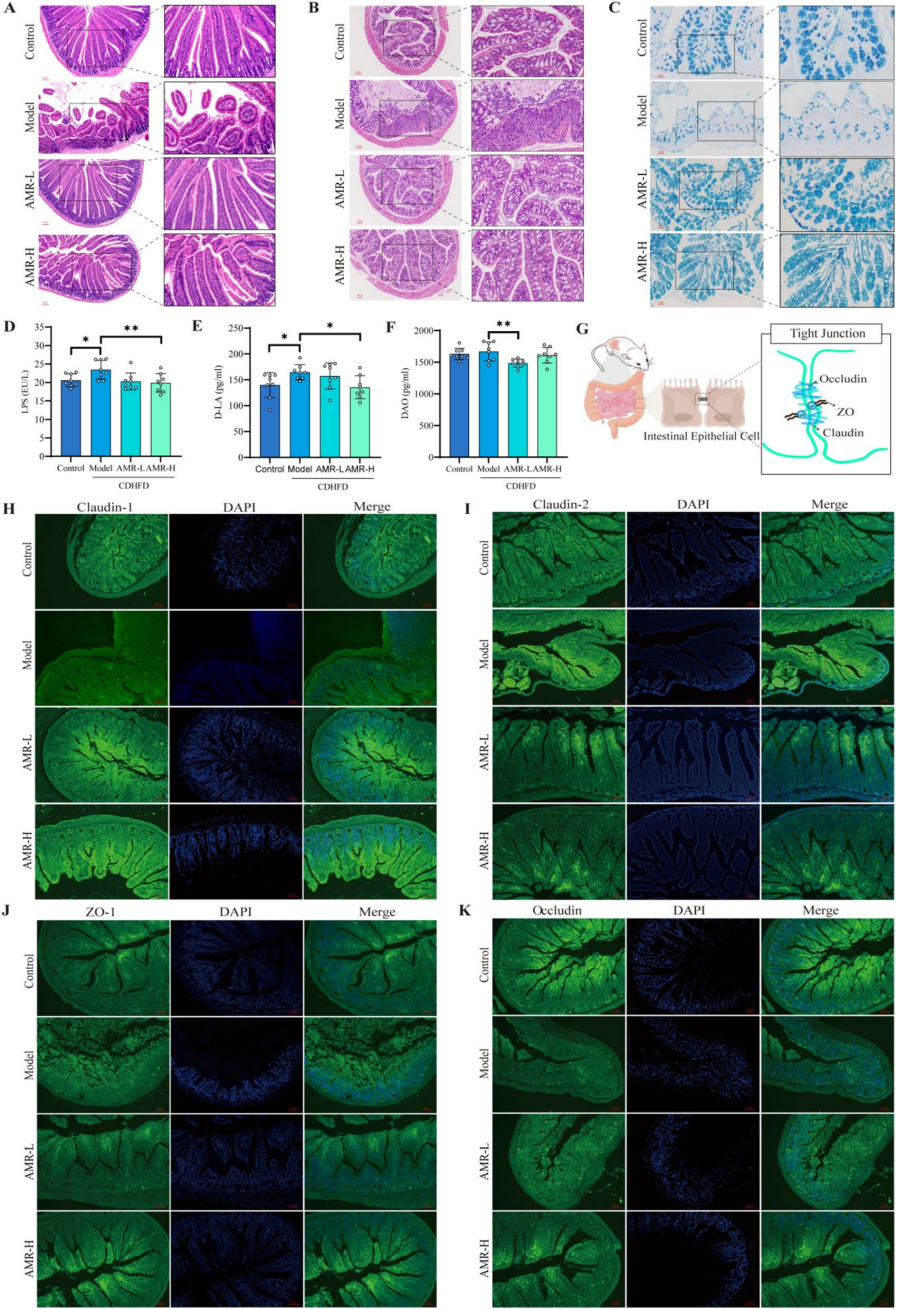
Effect of AMR on the intestinal mechanical barrier. **A**, **B** Representative H&E staining images of the duodenum (**A**) and colon (**B**) (magnification: 100 ×). **C** Representative AB-PAS staining images of the colon (magnification: 200 ×). **D**–**F** Serum levels of LPS (**D**), D-LA (**E**), and DAO (**F**). **G** Schematic diagram of intestinal epithelial tight junctions. **H**–**K** Representative immunofluorescence (IF) images showing the expression of Claudin-1 (**H**), Claudin-2 (**I**), ZO-1 (**J**), and Occludin (**K**) in the ileum (magnification: 200 ×). Data are presented as mean ± SD, n = 8. Statistical significance was evaluated using one-way ANOVA (followed by LSD or Dunnett’s T3 post-hoc tests) or the Kruskal–Wallis test. * *P* < 0.05, ** *P* < 0.01

H&E staining of the small intestine indicated that the intestinal villi in the control group were well-organized, elongated, and structurally intact. The epithelial cells lining the villi appeared as regular columnar cells without any visible lesions. Conversely, in the model group, the intestinal glands were disorganized and atrophied. The villi were severely damaged, showing blunting, structural fragmentation, loose arrangement, and localized fusion. Following AMR intervention, these pathological changes were effectively ameliorated. In the AMR-L group, the intestinal glands were neatly arranged, and the villi were restored to a prominent, tall, and columnar structure. In the AMR-H group, the recovery was even more pronounced, with densely and regularly arranged villi and tightly organized glands (Fig. 7A).

Similarly, H&E staining of the colon revealed that the colonic mucosa in the control group was structurally intact with a smooth surface, tightly arranged crypts, abundant goblet cells, and no signs of inflammatory cell infiltration. In the model group, CDHFD induced significant pathological alterations, including crypt atrophy and shortening, depletion of goblet cells, mucosal necrosis and detachment, and severe inflammatory cell infiltration. Notably, AMR intervention significantly alleviated these colonic damages (Fig. 7B).

Goblet cells synthesize and secrete mucins, which are crucial for maintaining the intestinal barrier function [34]. H&E staining indicated a notable depletion of goblet cells in the model group, which was reversed by AMR intervention. To further validate this, AB-PAS staining was utilized to evaluate the morphological and secretory functions of goblet cells. In the control group, goblet cells were abundant, well-organized, and exhibited robust secretion of acidic mucins. In stark contrast, the model group showed a significant reduction in goblet cell numbers, disorganized structural arrangement, cellular shrinkage, and severely depleted acidic mucin secretion. Following AMR treatment, the goblet cell population was successfully restored, their orderly arrangement was re-established, and the secretion of acidic mucins became highly abundant (Fig. 7C).

An improper diet can impair the functioning of the intestinal mechanical barrier, enhancing the permeability of the intestinal mucosa. This alteration permits the systemic translocation of gut-derived macromolecules (such as LPS and D-LA) into the bloodstream, and concurrently triggers the release of intracellular enzymes (such as DAO) from damaged intestinal mucosal cells [35–38]. Notably, in this context, serum LPS was evaluated primarily as a well-recognized surrogate marker for upstream macromolecular leakage (endotoxemia) rather than a mere inflammatory trigger. Our findings demonstrated that serum levels of LPS (Fig. 7D; *P* < 0.05) and D-LA (Fig. 7E; *P* < 0.05) were considerably elevated in the model group. AMR treatment, particularly high-dose AMR (AMR-H), significantly reduced these levels (Fig. 7D, E; *P* < 0.05 or *P* < 0.01). Furthermore, elevated DAO levels were significantly suppressed in the AMR-L group (Fig. 7F; *P* < 0.01). Collectively, these indicators confirm that AMR effectively restores intestinal permeability and prevents CDHFD-induced gut-derived macromolecular translocation.

As shown in Fig. 7G, intestinal epithelial cell tight junctions are the primary connection between intestinal epithelial cells and play an important role in maintaining cell polarity and regulating intestinal permeability [39]. To elucidate the molecular mechanisms underlying AMR-mediated barrier protection, the protein expression levels of tight junction components (Claudin-1, ZO-1, Occludin, and Claudin-2) in ileal tissues were investigated (Fig. 7G). Compared with the control group, the expression levels of the barrier-forming proteins Claudin-1 (Fig. 7H), ZO-1 (Fig. 7J), and Occludin (Fig. 7K) were significantly downregulated, whereas the level of the pore-forming protein Claudin-2 (Fig. 7I) was upregulated in the model group. AMR treatment effectively reversed these trends, significantly upregulating Claudin-1, ZO-1, and Occludin, while downregulating Claudin-2, with the most robust therapeutic effects observed in the AMR-H group. These results indicate that AMR successfully repairs the intestinal mucosal barrier by modulating tight junction protein expression.

### Effect of AMR on the TRAF6/A20/NF-κB inflammatory signaling axis

LPS serves as a significant contributor to systemic inflammation, facilitating the activation and infiltration of inflammatory cells while amplifying both the severity and duration of the inflammatory response. The ability of LPS to induce inflammation largely relies on its capacity to enter the bloodstream. Intestinal barrier damage abnormally increases paracellular permeability, allowing LPS to translocate into the systemic circulation, activate the NF-κB signaling pathway, and trigger inflammation by stimulating pro-inflammatory cytokine production [40]. Consistently, we found that CDHFD significantly elevated serum LPS levels and induced an inflammatory response, which was attenuated after AMR intervention. To further assess how AMR influences these inflammatory pathways, we measured the expression levels of key NF-κB regulatory proteins, including A20, TRAF6, IκB, and p65 in colon tissues.

**Fig. 8:**
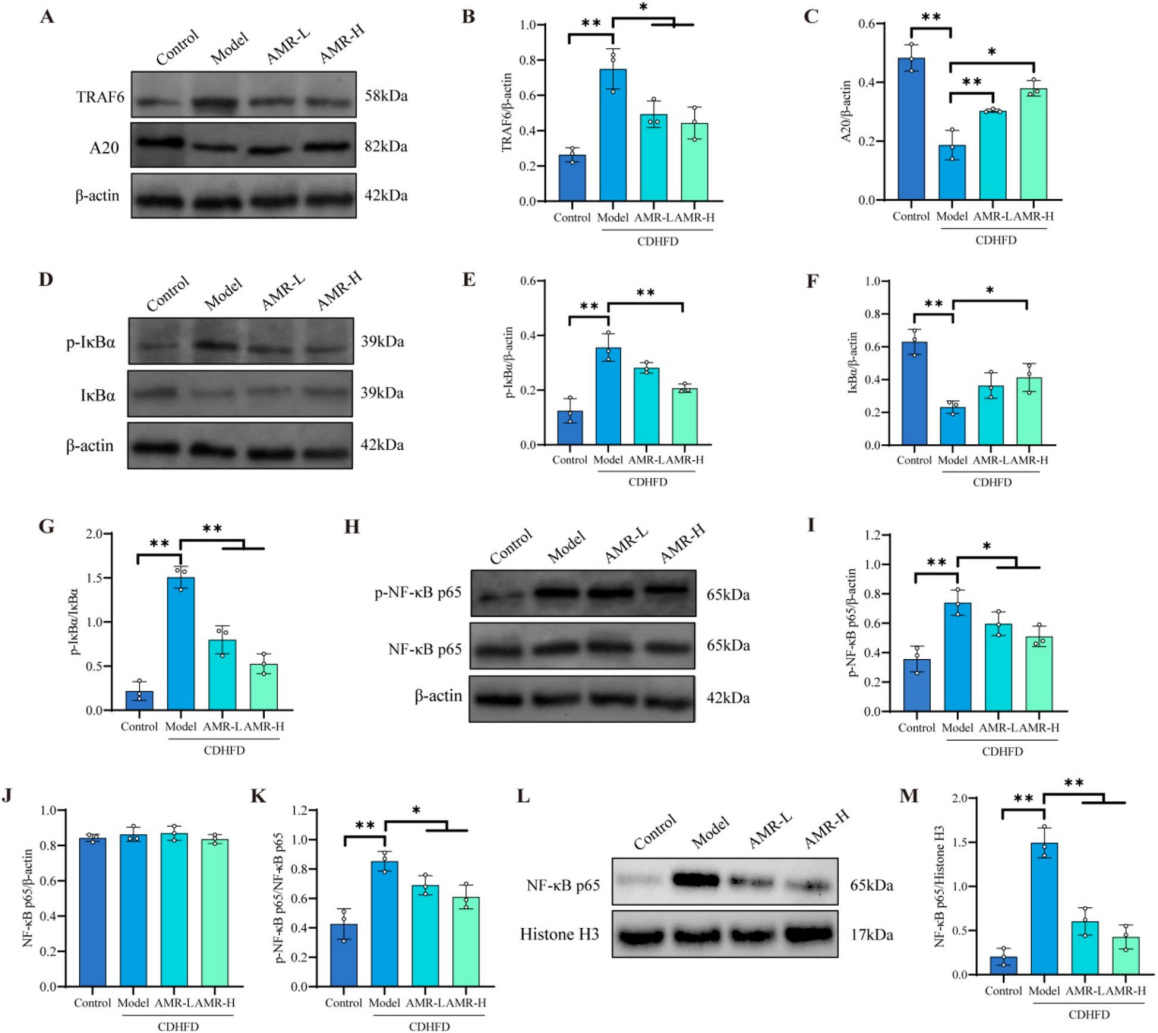
Effect of AMR on the TRAF6/A20/NF-κB inflammatory signaling axis. **A**–**C** Representative Western blot images and quantitative analysis of relative protein expression levels of TRAF6 and A20. **D**–**G** Representative Western blot images and quantitative analysis of p-IκBα, IκBα, and the p-IκBα/IκBα ratio. **H**–**K** Representative Western blot images and quantitative analysis of p-NF-κB p65, NF-κB p65, and the p-p65/p65 ratio. **L**, **M** Representative Western blot images and quantitative analysis showing the protein levels of NF-κB p65 in nuclear fractions. The relative expression levels of total proteins were normalized to β-actin, while nuclear p65 was normalized to Histone H3. Data are presented as mean ± SD, n = 3. Statistical significance was evaluated using one-way ANOVA (followed by LSD or Dunnett’s T3 post hoc tests) or the Kruskal–Wallis test. * *P* < 0.05, ** *P* < 0.01

Our findings indicate that TRAF6 levels were significantly elevated in the model group but markedly suppressed following AMR intervention (Fig. 8A, B; *P* < 0.05 *or P* < 0.01). Crucially, the protein level of A20, a key negative regulator of NF-κB, was significantly decreased in the model group but markedly restored following AMR intervention (Fig. 8A, C; *P* < 0.05 *or P* < 0.01). This indicates that AMR effectively enhances A20-mediated inhibitory feedback. Regarding the IκB/NF-κB axis, the model group exhibited substantial IκBα degradation, characterized by decreased total IκBα, increased *p*-IκBα, and an elevated *p*-IκBα/IκBα ratio. In contrast, AMR treatment reversed these trends (Fig. 8D–G; *P* < 0.05 *or P* < 0.01). Although total NF-κB p65 levels remained unchanged across all groups (Fig. 8H, J; *P* > 0.05), AMR significantly attenuated the CDHFD-induced phosphorylation of p65 and reduced the *p*-p65/p65 ratio (Fig. 8H, I, K; *P* < 0.05 *or P* < 0.01). Furthermore, the robust nuclear accumulation of NF-κB p65 observed in the intestines of the model group was markedly prevented by AMR intervention (Fig. 8L, M; *P* < 0.01). Collectively, these findings demonstrate that AMR inhibits the excessive activation and nuclear translocation of the NF-κB signaling pathway.

## Discussion

The global shift toward modern dietary patterns is increasingly characterized by the concurrent consumption of high-fat foods and cold beverages. Although highly prevalent, this specific dietary combination imposes severe physiological stress on the gastrointestinal tract [41–43]. Substantial evidence indicates that excessive intake of dietary lipids not only significantly increases the incidence of functional diarrhea but also predisposes individuals to broader gastrointestinal disorders. Concurrently, cold thermal stimulation from iced drinks has been shown to exacerbate intestinal hypersensitivity and trigger diarrheal episodes, particularly in individuals with irritable bowel syndrome or compromised gastrointestinal function [44]. To investigate the underlying pathophysiological mechanisms of this modern phenomenon, our previous work successfully established a novel mouse model induced by CDHFD. This regimen effectively mimics unhealthy contemporary eating habits and accurately recapitulates human dietary-induced diarrhea, which is intrinsically accompanied by profound gastrointestinal dysmotility, exacerbated systemic and local inflammation, and severe impairment of the intestinal mucosal barrier [8].

Currently, the clinical management of diarrhea predominantly relies on symptomatic pharmacological interventions, such as loperamide to inhibit intestinal motility, octreotide to regulate gastrointestinal hormones, or fluoroquinolone antibiotics for infectious etiologies. Although these agents offer rapid symptomatic relief, they often fail to address the underlying pathogenesis. Consequently, enhancing the inherent defense and regenerative capacities of the intestinal mucosa is paramount for long-term therapeutic efficacy. Traditional Chinese Medicine offers a holistic paradigm for managing gastrointestinal disorders. Specifically, AMR, a classic botanical drug traditionally utilized to invigorate the spleen and alleviate diarrheal symptoms, has been validated by modern pharmacological studies to exert pleiotropic effects, including modulating gut microbiota, protecting the intestinal epithelium, and mitigating inflammation [45–47]. Consistent with these properties, our present study demonstrated that AMR intervention robustly ameliorated CDHFD-induced functional diarrhea (Fig. 4A–D). Notably, chronic and recurrent diarrhea is not merely a manifestation of altered motility, but is intrinsically linked to a sustained pathological cycle driven by a compromised intestinal barrier and uncontrolled inflammatory cascades. The intestinal barrier, serving as the paramount frontline defense against luminal pathogens and toxins, is indispensable for maintaining intestinal homeostasis [48, 49]. Our integrated analyses comprehensively reveal that the therapeutic efficacy of AMR extends beyond superficial symptom relief; it functions through a multi-target mechanism encompassing the restructuring of dysbiotic gut microbiota (Figs. 5, 6), the restoration of intestinal barrier integrity (Fig. 7), and the profound suppression of systemic and colonic inflammation (Fig. 4).

Recent studies have increasingly highlighted the indirect pharmacological paradigms of AMR, demonstrating that its active polysaccharides predominantly target the gut microbiota to generate beneficial metabolites (e.g., modulating tryptophan metabolism) rather than relying on direct systemic absorption. Gut microbiota dysbiosis serves as a crucial driver in exacerbating aberrant inflammatory and immune responses through multiple pathways [50, 51]. This perturbation fuels a vicious cycle of local intestinal inflammation and is intrinsically linked to the pathogenesis and progression of diverse enteric disorders [43, 52, 53]. In concordance with this, our results indicate that CDHFD exposure elicited profound shifts in both the composition and structure of the gut microbiome, whereas AMR intervention effectively ameliorated these dysbiotic alterations. Although our findings primarily highlight a microbiota-to-barrier mechanistic cascade, the host-microbiome interaction is inherently bidirectional. Just as dysbiosis impairs the barrier, restoring barrier integrity reversely reshapes the gut microbiota by providing a favorable niche for commensals, thereby breaking the vicious cycle of dysbiosis. This bidirectional modulation aligns with recent literature, which demonstrates that AMR exhibits potent prebiotic-like effects by enriching beneficial taxa and maintaining intestinal homeostasis across various disease models [54–57].

Research has shown that the abundance of *norank_f_Muribaculaceae* is significantly diminished in several disease states, including irritable bowel syndrome and ulcerative colitis. This taxon is a pivotal producer of short-chain fatty acids (SCFAs), such as acetate and propionate, which are vital for maintaining intestinal barrier integrity and reducing inflammation [58]. Furthermore, it may directly facilitate mucosal repair by promoting mucin secretion [59], and can interact synergistically with traditional probiotics like *Bifidobacterium* and *Lactobacillus* to promote broader intestinal health [60]. Similarly, the *Lachnospiraceae_NK4A136_group* is a critical butyrate-producing keystone taxon whose depletion is strongly correlated with IBS-D and other enteric disorders [61]. The loss of this bacterium deprives colonic epithelial cells of their primary energy source derived from dietary fiber fermentation, thereby critically compromising mucosal barrier function and amplifying susceptibility to inflammation [62]. Crucially, our findings demonstrate that CDHFD significantly depleted the populations of both *norank_f_Muribaculaceae* and the *Lachnospiraceae_NK4A136_group*, while AMR intervention robustly enriched these protective, SCFA-producing taxa (Fig. 6D, E).

Spearman correlation analysis further revealed significant associations between distinct gut microbial taxa and clinical indicators. Specifically, the abundance of *norank_f_Muribaculaceae* was strongly negatively correlated with systemic inflammatory indices, including pro-inflammatory cytokines (IL-6, IL-1β, and TNF-α) and lymphocyte counts, while the *Lachnospiraceae_NK4A136_group* exhibited a substantial negative correlation with serum LPS levels (Fig. 6G). Collectively, these correlations suggest a potential mechanistic link wherein these specific taxa mitigate systemic inflammation by facilitating the repair of the damaged intestinal barrier.

To further elucidate the indirect pharmacological mechanism by which AMR treats CDHFD-induced diarrhea, PICRUSt2 functional prediction was employed to reveal shifts in the microbial metabolic landscape. Notably, we observed a significant enrichment in terminal pathways for short-chain fatty acid (SCFA) biosynthesis, specifically “butanoate metabolism” and “propanoate metabolism”. This was accompanied by a marked increase in the predicted abundance of upstream SCFA precursors and carbohydrate fermentation pathways, such as “pyruvate metabolism” and “starch and sucrose metabolism”. The robust enrichment of these upstream metabolic engines alongside terminal synthesis pathways suggests that the therapeutic efficacy of AMR is closely linked to an enhanced microbial capacity for SCFA production. It is well-established that SCFAs, particularly butyrate, serve as the primary energy source for colonocytes and are indispensable for stimulating MUC2 mucin secretion, maintaining tight junction integrity, and dampening mucosal inflammation [63]. Therefore, this enhanced SCFA-producing potential strongly supports our hypothesis that AMR alleviates intestinal barrier damage and diarrhea indirectly by remodeling the functional output of the gut microbiome.

One of the primary mechanisms involved in the onset and progression of intestinal diseases is chronic low-grade inflammation related to the activation of the immune system, a phenomenon that is closely connected to the compromised integrity of the intestinal barrier [64, 65]. The intestinal barrier effectively protects the body against luminal microorganisms and toxins. When its function is impaired, abnormal openings in the paracellular pathways of intestinal epithelial cells increase intestinal permeability, allowing macromolecules such as LPS to readily translocate into the systemic circulation, thereby triggering inflammation [48, 66]. Concurrently, inflammation disrupts the tight junctions between intestinal epithelial cells, further exacerbating intestinal barrier damage [67, 68]. In our study, CDHFD significantly downregulated sealing tight junction proteins (Occludin, ZO-1, and Claudin-1) while upregulating the pore-forming protein Claudin-2, coinciding with elevated serum levels of D-LA and DAO. Notably, AMR intervention effectively restored these tight junction profiles and mitigated intestinal permeability (Fig. 7E–K). As a macromolecular substance, abnormally increased intestinal permeability is considered a major route that facilitates LPS translocation into the bloodstream. We found that the serum LPS levels in model mice were significantly elevated, a phenomenon that was reversed by AMR intervention (Fig. 7D). These results suggest that LPS likely enters the circulation via a compromised intestinal barrier. Once in the blood, LPS triggers inflammation by activating the NF-κB signaling pathway in a TRAF6-dependent manner [69]. Mechanistically, we found that AMR significantly downregulated the expression of the upstream adaptor protein TRAF6 (Fig. 8). More importantly, AMR treatment significantly upregulated the expression of A20, a well-known negative regulator of NF-κB signaling. Given that A20 typically restricts inflammatory responses by targeting proteins like TRAF6, our findings suggest that AMR promotes the resolution of inflammation through an A20-mediated inhibitory feedback loop, thereby effectively suppressing the excessive activation of the downstream NF-κB pathway.

**Fig. 9:**
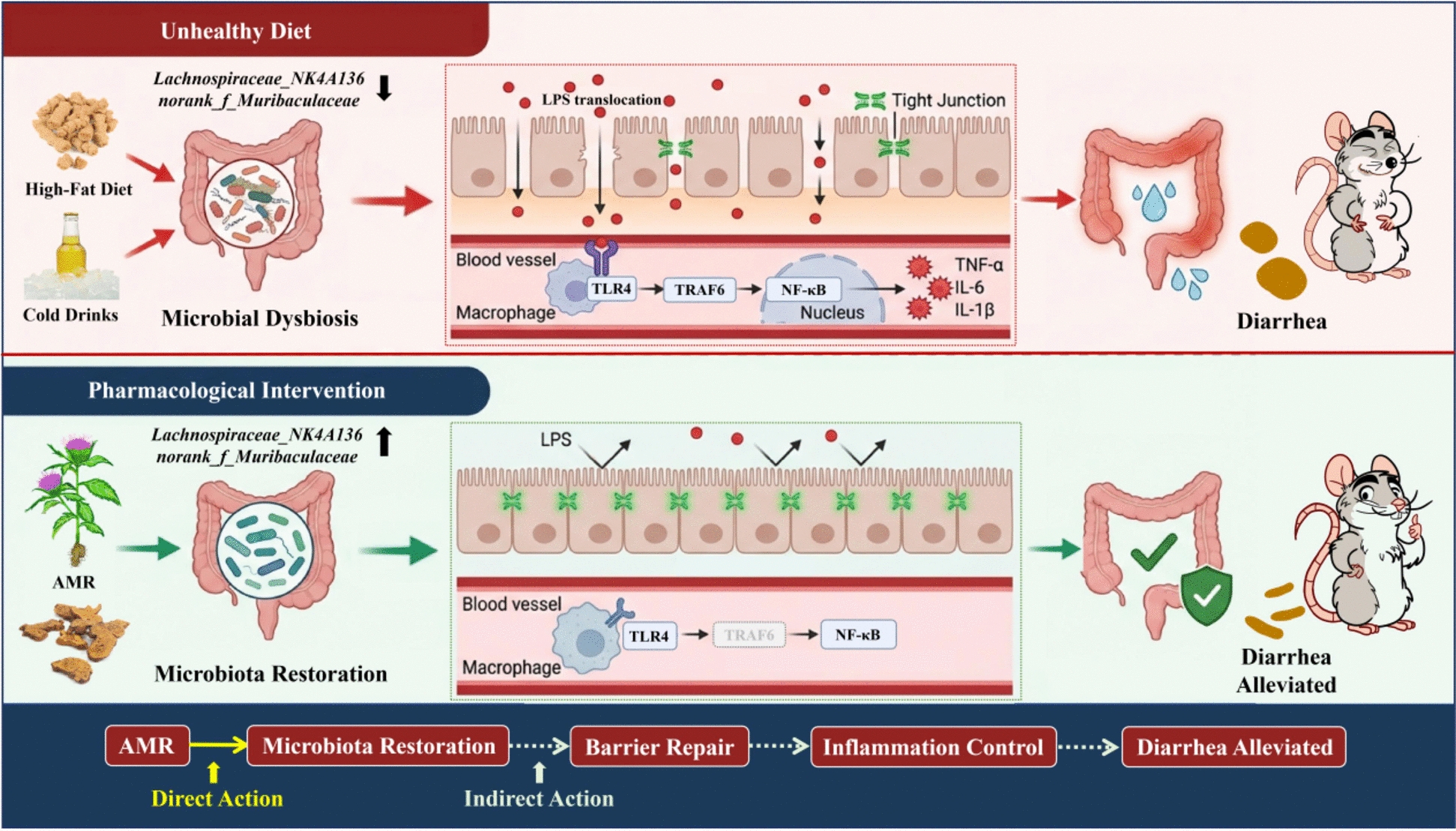
Schematic diagram illustrating the mechanism by which AMR ameliorates CDHFD-induced diarrhea

Collectively, we propose that AMR ameliorates diarrhea primarily by orchestrating gut microbiota remodeling rather than by directly targeting host organs. Specifically, AMR significantly enriches beneficial bacterial taxa (e.g., *Lachnospiraceae_NK4A136_group*), which facilitates the restoration of tight junction proteins (such as ZO-1) and reinforces intestinal barrier integrity, as evidenced by reduced serum D-LA levels. This barrier restoration effectively impedes the systemic translocation of luminal LPS, thereby dampening systemic inflammation, as evidenced by the reduction of pro-inflammatory cytokines like IL-6. In summary, our study demonstrates that AMR functions via a microbiota-barrier-inflammation axis, blocking the LPS/NF-κB signaling cascade to ultimately alleviate systemic inflammation and diarrhea (Fig. 9).

Although this study demonstrates that the antidiarrheal effect of AMR is largely mediated by modulation of the gut microbiota, several limitations warrant further investigation. Firstly, the specific active constituents of AMR and the precise host-microbiome interactions remain incompletely defined. Given that the core efficacy of AMR relies on indirect pharmacological actions, specifically the regulation of the gut microecology and the intestinal barrier, the observed therapeutic effects cannot yet be attributed to a single bioactive compound. Therefore, future studies integrating fecal microbiota transplantation (FMT) or gut microbiota-cell co-culture systems, alongside pharmacokinetic and metabolomic analyses, are required to pinpoint the primary active substances and their specific molecular targets. Secondly, while this study focused heavily on the modern molecular and microecological mechanisms of AMR against CDHFD-induced diarrhea, we did not systematically record all the classic macroscopic manifestations of Spleen deficiency in TCM theory, such as fur condition, food intake fluctuations, and specific perianal soiling degree. Future studies should incorporate standardized TCM syndrome scoring systems to provide a more comprehensive evaluation of AMR’s efficacy. Thirdly, regarding the CDHFD model, while cold beer was employed to simulate the prevalent modern dietary habit of pairing high-fat diets with cold alcoholic beverages, the confounding effect of alcohol must be acknowledged. Because alcohol independently alters intestinal permeability and microbiota composition, the distinct contributions of cold stress versus mild alcohol exposure to the observed barrier impairment require further clarification using a plain cold-water control group in future investigations. Finally, our current findings primarily establish correlations, rather than direct causal relationships, among microbiota modulation, barrier restoration, and reduced LPS translocation. To definitively elucidate the causal mechanisms underlying the microbiota-barrier-inflammation axis, future validation studies employing FMT, FITC-dextran gavage (to directly assess in vivo permeability), Caco-2 cell monolayers, and TLR4 knockout mice are highly warranted.

AMR exerts its therapeutic effects via the microbiota-barrier-inflammation axis. Specifically, AMR-induced remodeling of the gut microbiota facilitates intestinal barrier repair, which in turn prevents the systemic translocation of luminal LPS. This blockade, coupled with the upregulation of the A20-mediated negative feedback loop, effectively suppresses the TRAF6/NF-κB signaling cascade. Consequently, systemic inflammation is attenuated, ultimately alleviating CDHFD-induced diarrhea. (Solid arrows indicate direct interactions; dashed arrows indicate microbiota-mediated indirect cascades).

## Conclusion

In conclusion, this study demonstrates that AMR effectively ameliorates CDHFD-induced diarrhea through a microecology-dependent mechanism. Rather than directly targeting host organs, AMR exerts its therapeutic effects by modulating the gut microbiota-barrier-inflammation axis. By restoring intestinal microbial homeostasis, AMR fortifies the intestinal mechanical barrier, thereby preventing endotoxin-driven systemic immune hyperactivation. Collectively, these findings provide experimental evidence for the potential clinical application of AMR as a microbiota-targeted therapy for diarrhea.

## Supplementary Information

Supplementary Material 1.

Supplementary Material 2.

## Data Availability

The data produced from this study are available from the corresponding author on reasonable request.

## Abbreviations

AMR: Atractylodis Macrocephalae Rhizoma
A20: TNFAIP3 (Tumor Necrosis Factor Alpha-Induced Protein 3)
AB-PAS: Alcian Blue-Periodic Acid Schiff
CDHFD: Cold drinks and high-fat diet
DAO: Diamine oxidase
d-LA: D-Lactic acid
5-HT: 5-Hydroxytryptamine
FMT: Fecal microbiota transplantation
FITC: Fluorescein isothiocyanate
GAS: Gastrin
H&E: Hematoxylin and eosin
HPLC: High-performance liquid chromatography
IκB: Inhibitor of nuclear factor kappa B
IL-1: Interleukin-1
IL-6: Interleukin-6
IgG: Immunoglobulin G
IF: Immunofluorescence
KS: Kolmogorov–Smirnov
KW: Kruskal–Wallis
LPS: Lipopolysaccharide
MTL: Motilin
NF-κB: Nuclear Factor Kappa B
PCA: Principal component analysis
PLS-DA: Partial Least Squares Discriminant Analysis
RDA: Redundancy analysis
TCMSP: Traditional Chinese Medicine Systems Pharmacology
TNF-α: Tumor necrosis factor-alpha
TRAF6: Tumor Necrosis Factor Receptor-Associated Factor 6
TCM: Traditional Chinese Medicine
TLR4: Toll-like receptor 4
UPLC-ESI-MS: Ultra-performance liquid chromatography-electrospray ionization-mass spectrometry
VIP: Vasoactive intestinal peptide
ZO-1: Zonula occludens-1
