# Supplementary material for: Atractylodis Macrocephalae Rhizoma ameliorates diarrhea induced by cold drinks and a high-fat diet by remodeling gut microecology and restoring barrier function
Source: Chin Med. 2026 Jul 15;21:191. doi: 10.1186/s13020-026-01467-0 (PMC13371211; doi:10.1186/s13020-026-01467-0)
Supplement: Supplementary file 1 — Supplementary Material 1. [file 13020_2026_1467_MOESM1_ESM.pdf]

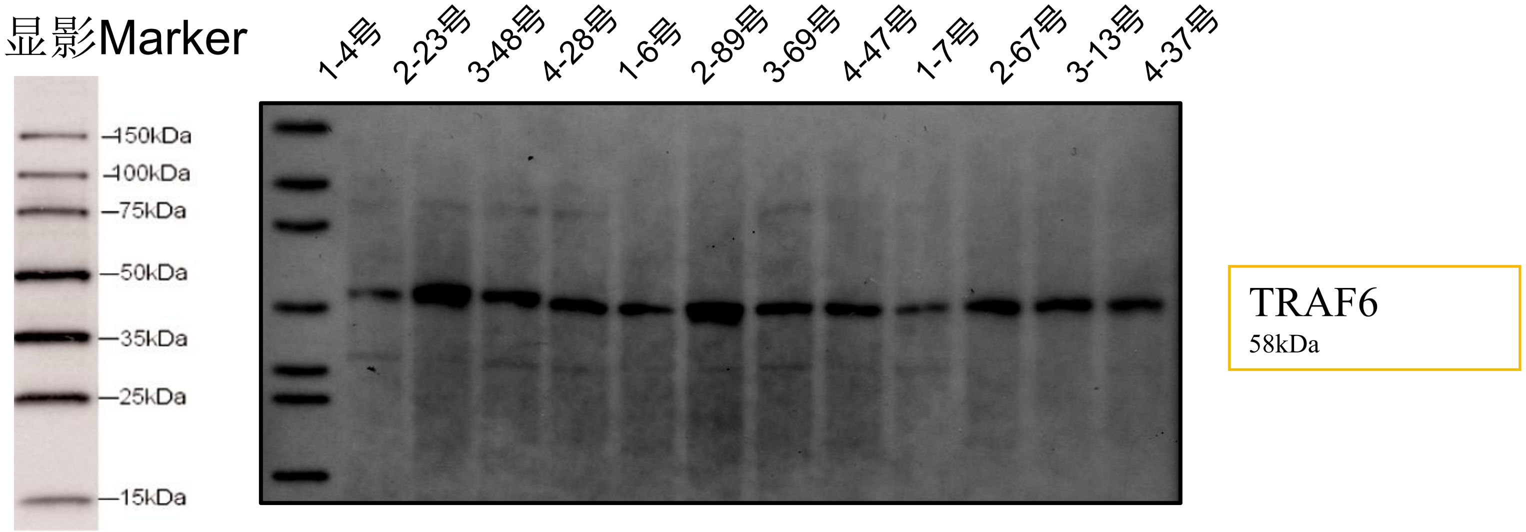
显影Marker
150kDa
100kDa
75kDa
50kDa
35kDa
25kDa
15kDa
1-4号
2-23号
3-48号
4-28号
1-6号
2-89号
3-69号
4-47号
1-7号
2-67号
3-13号
4-37号
TRAF6
58kDa

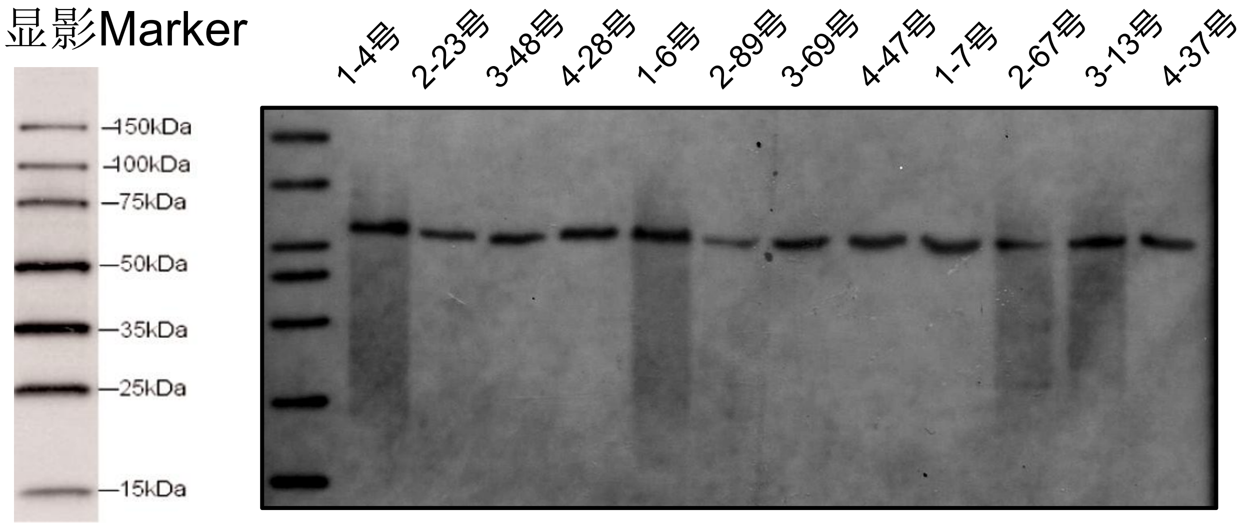

显影Marker
450kDa
400kDa
75kDa
50kDa
35kDa
25kDa
15kDa
1-4号
2-23号
3-48号
4-28号
1-6号
2-89号
3-69号
4-47号
1-7号
2-67号
3-13号
4-37号

A20
82kDa

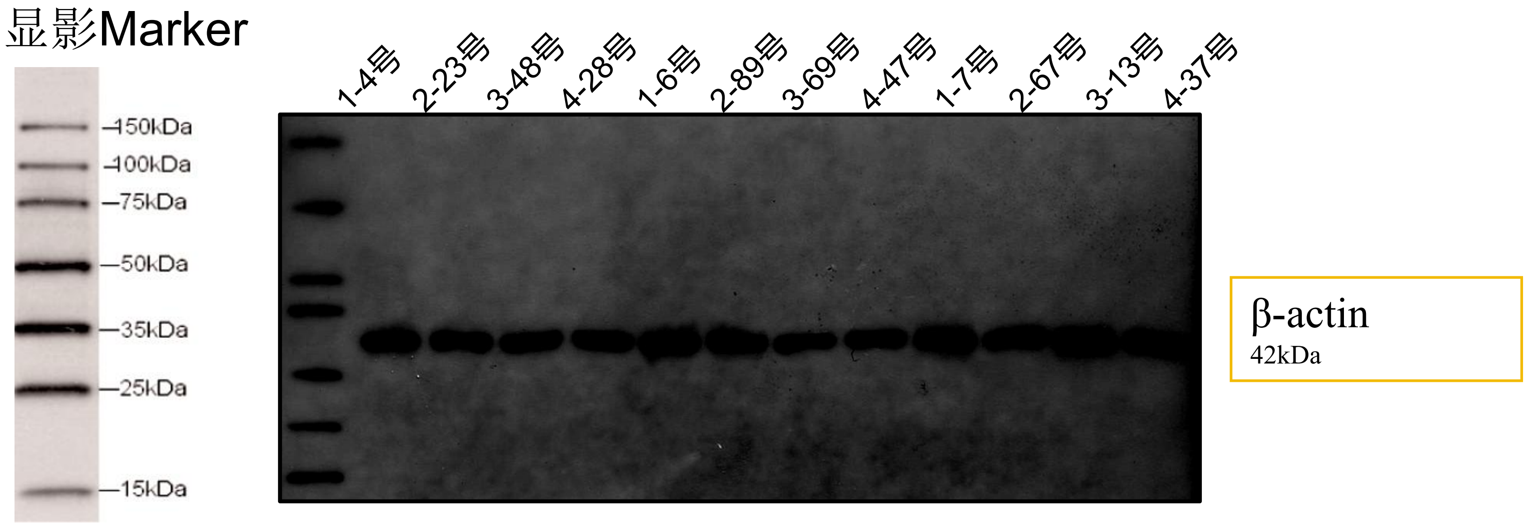
显影Marker
450kDa
400kDa
75kDa
50kDa
35kDa
25kDa
15kDa
1-4号
2-23号
3-48号
4-28号
1-6号
2-89号
3-69号
4-47号
1-7号
2-67号
3-13号
4-37号
β-actin
42kDa

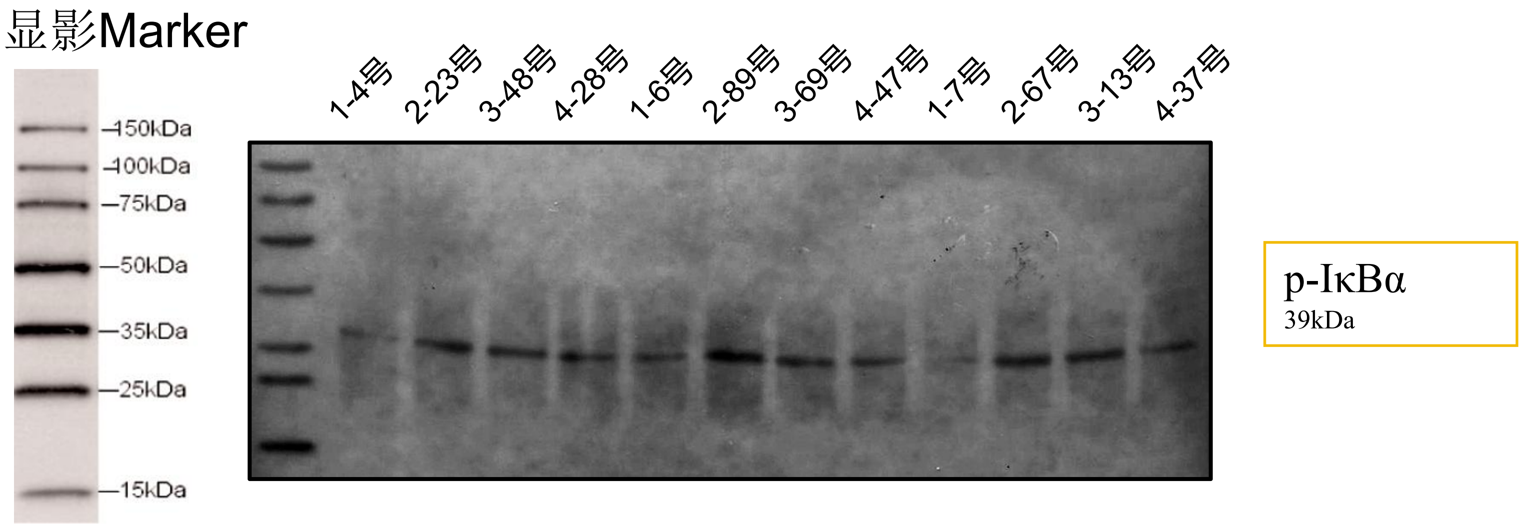

显影Marker
150kDa
100kDa
75kDa
50kDa
35kDa
25kDa
15kDa
1-4号
2-23号
3-48号
4-28号
1-6号
2-89号
3-69号
4-47号
1-7号
2-67号
3-13号
4-37号
p-IκBα
39kDa

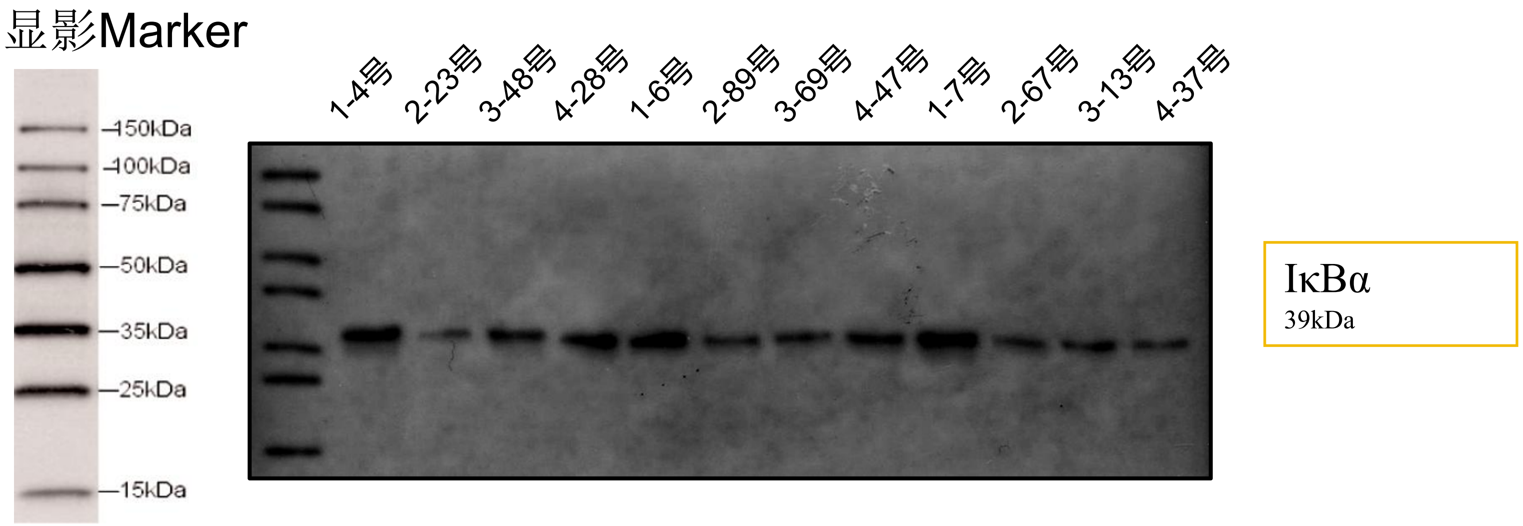

显影Marker
150kDa
100kDa
75kDa
50kDa
35kDa
25kDa
15kDa
1-4号
2-23号
3-48号
4-28号
1-6号
2-89号
3-69号
4-47号
1-7号
2-67号
3-13号
4-37号
IκBα
39kDa

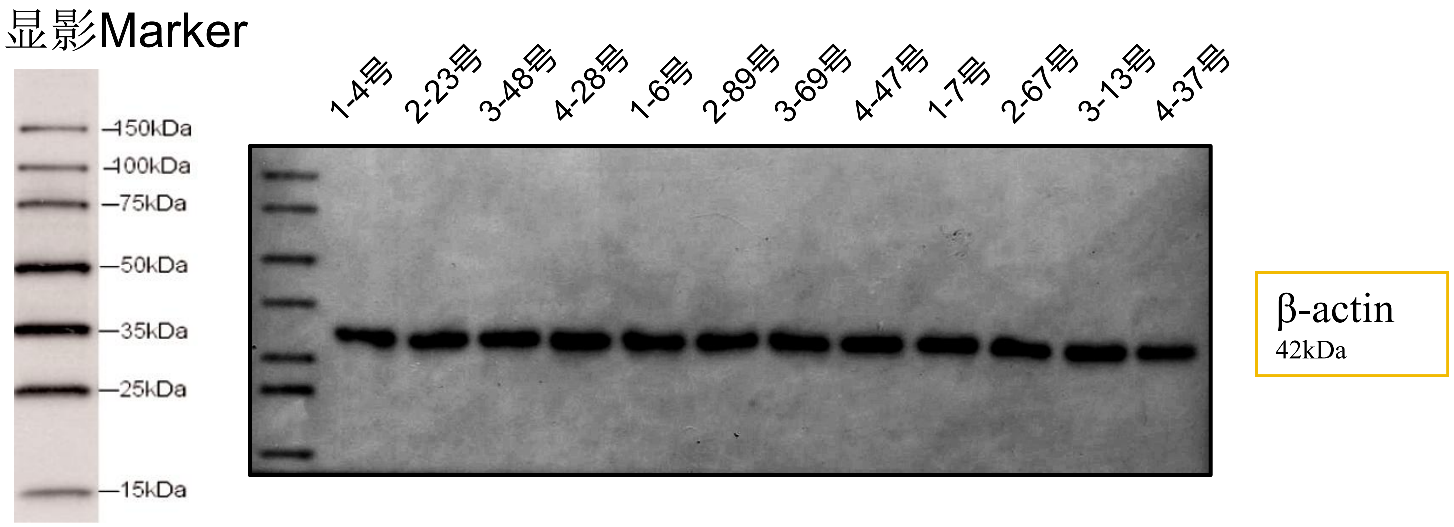
显影Marker
150kDa
100kDa
75kDa
50kDa
35kDa
25kDa
15kDa
1-4号
2-23号
3-48号
4-28号
1-6号
2-89号
3-69号
4-47号
1-7号
2-67号
3-13号
4-37号
β-actin
42kDa

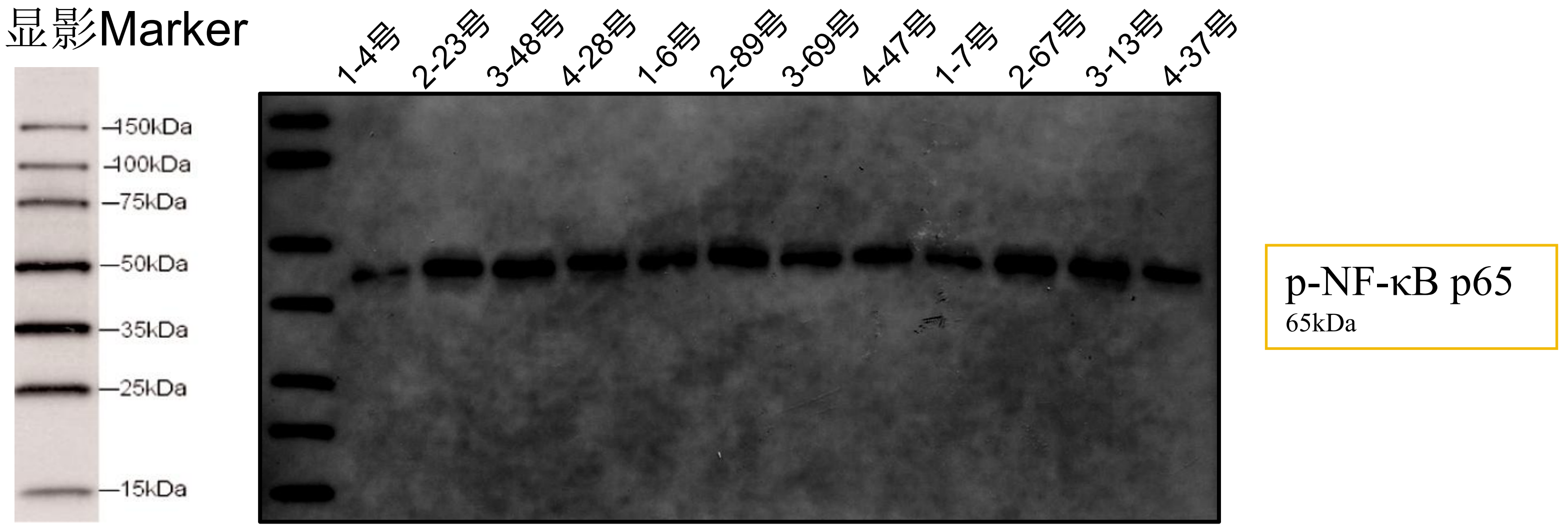

显影Marker
450kDa
100kDa
75kDa
50kDa
35kDa
25kDa
15kDa
1-4号
2-23号
3-48号
4-28号
1-6号
2-89号
3-69号
4-47号
1-7号
2-67号
3-13号
4-37号
p-NF-κB p65
65kDa

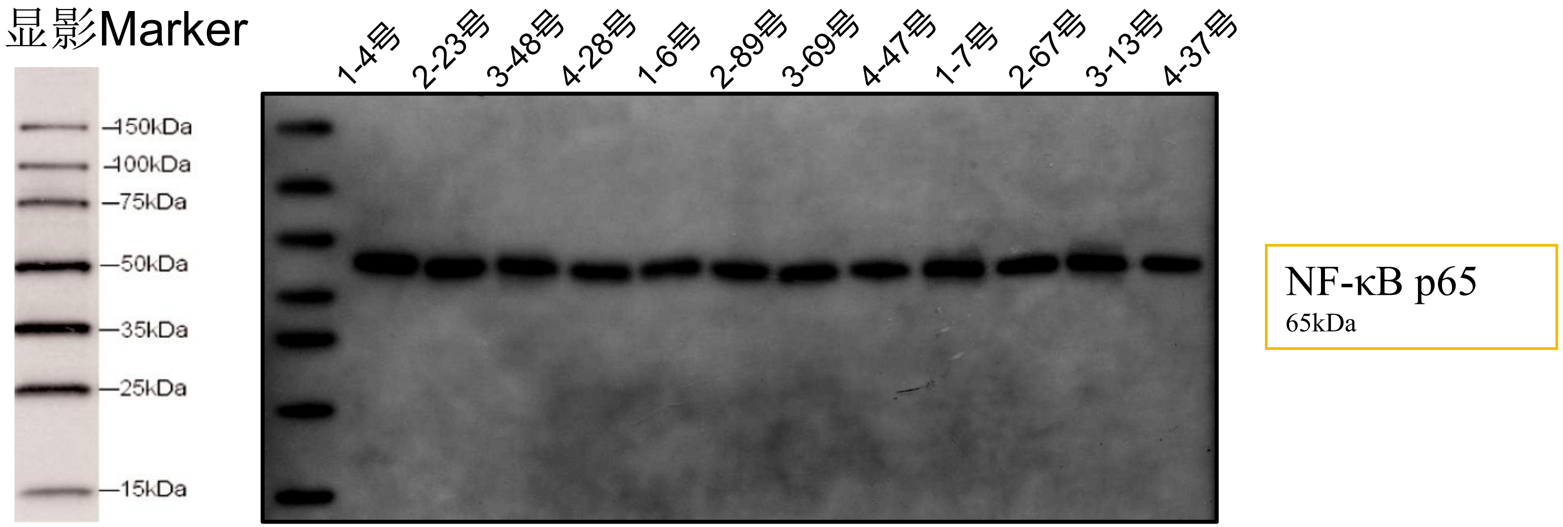

显影Marker
150kDa
100kDa
75kDa
50kDa
35kDa
25kDa
15kDa
1-4号
2-23号
3-48号
4-28号
1-6号
2-89号
3-69号
4-47号
1-7号
2-67号
3-13号
4-37号
NF-κB p65
65kDa

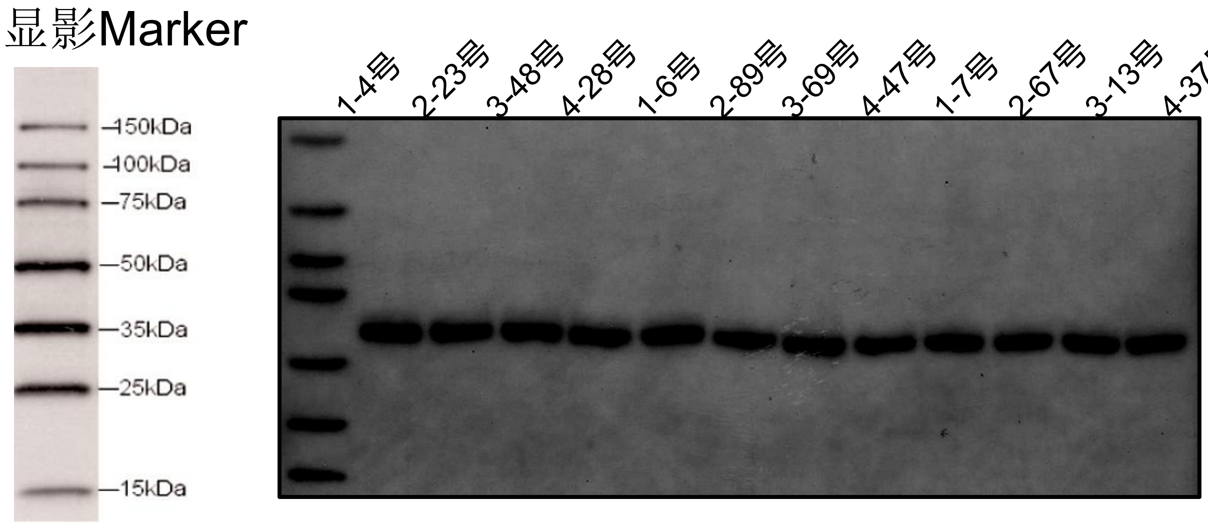

β-actin
42kDa

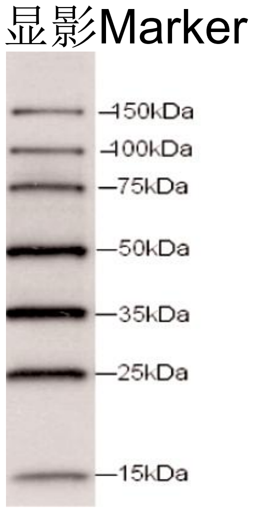
显影Marker
150kDa
100kDa
75kDa
50kDa
35kDa
25kDa
15kDa

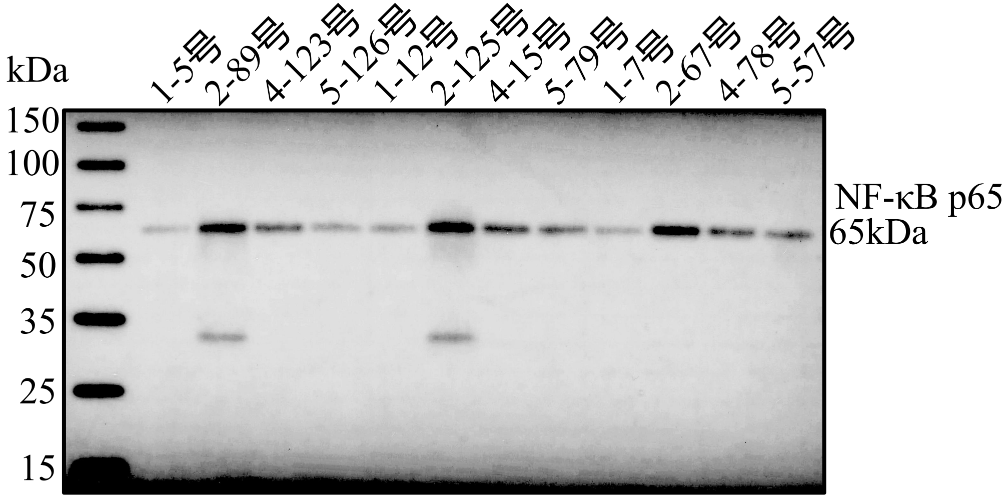
kDa
1-5号
2-89号
4-123号
5-126号
1-12号
2-125号
4-15号
5-79号
1-7号
2-67号
4-78号
5-57号
150
100
75
50
35
25
15
NF-κB p65
65kDa

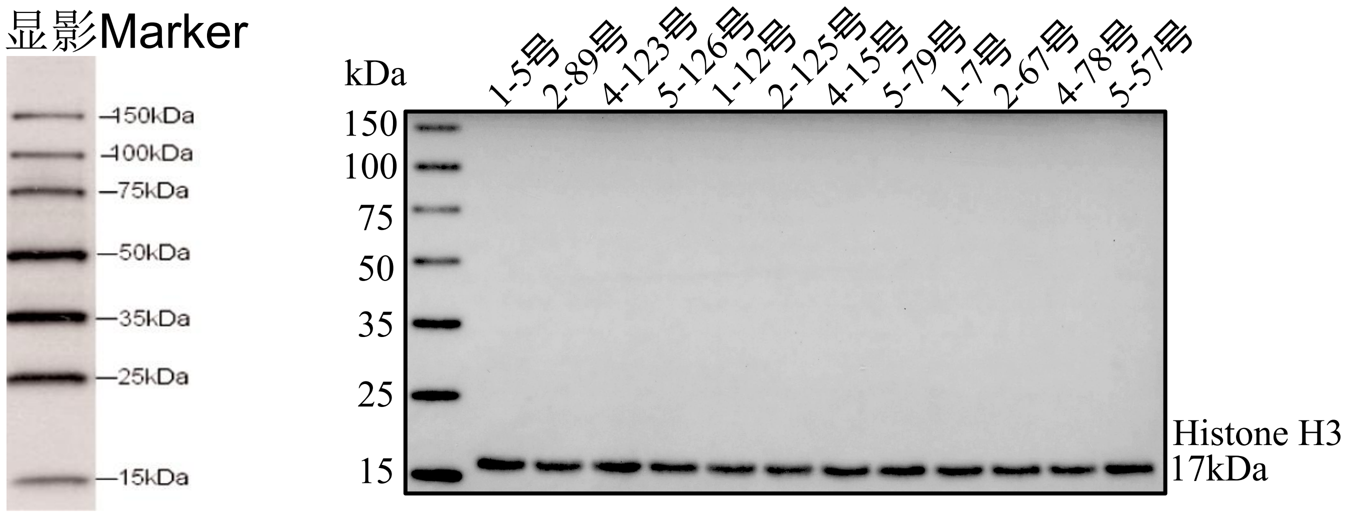

显影Marker
150kDa
100kDa
75kDa
50kDa
35kDa
25kDa
15kDa
kDa
1-5号
2-89号
4-123号
5-126号
1-12号
2-125号
4-15号
5-79号
1-7号
2-67号
4-78号
5-57号
150
100
75
50
35
25
15
Histone H3
17kDa
