# Supplementary material for: Atractylodis Macrocephalae Rhizoma ameliorates diarrhea induced by cold drinks and a high-fat diet by remodeling gut microecology and restoring barrier function
Source: Chin Med. 2026 Jul 15;21:191. doi: 10.1186/s13020-026-01467-0 (PMC13371211; doi:10.1186/s13020-026-01467-0)
Supplement: Supplementary file 2 — Supplementary Material 2. [file 13020_2026_1467_MOESM2_ESM.pdf]

加样孔	1	2	3	4	5	6	7	8	9	10	11	12
样品名称	1-4号	2-23号	3-48号	4-28号	1-6号	2-89号	3-69号	4-47号	1-7号	2-67号	3-13号	4-37号
TRAF6												
	3.81	11.22	8.54	7.57	4.32	11.71	6.30	6.29	3.11	8.83	6.54	4.92
	3.96	11.51	8.65	7.8	4.53	11.87	6.4	6.53	3.24	8.93	6.64	5.19
	4.22	11.81	8.75	7.95	4.7	12.04	6.61	6.71	3.51	9.04	6.78	5.44
β-actin												
	14.5	14.16	14.77	14.4	14.76	14.13	14.08	14.17	14.66	14.25	14.68	14.41
	14.79	14.37	14.93	14.66	14.91	14.35	14.27	14.41	14.78	14.51	14.96	14.66
	15.07	14.56	15.18	14.88	15.1	14.46	14.46	14.68	14.89	14.81	15.08	14.82
TRAF6/β-actin												
	0.26	0.79	0.58	0.53	0.29	0.83	0.45	0.44	0.21	0.62	0.45	0.34
	0.27	0.80	0.58	0.53	0.30	0.83	0.45	0.45	0.22	0.62	0.44	0.35
	0.28	0.81	0.58	0.53	0.31	0.83	0.46	0.46	0.24	0.61	0.45	0.37
Mean	0.27	0.80	0.58	0.53	0.30	0.83	0.45	0.45	0.22	0.62	0.45	0.35
Mean ± SD	0.27±0.01	0.8±0.01	0.58±0	0.53±0	0.3±0.01	0.83±0	0.45±0.01	0.45±0.01	0.22±0.01	0.62±0	0.45±0	0.35±0.01

加样孔	1	2	3	4	5	6	7	8	9	10	11	12
样品名称	1-4号	2-23号	3-48号	4-28号	1-6号	2-89号	3-69号	4-47号	1-7号	2-67号	3-13号	4-37号
A20												
	7.61	3.19	4.51	5.79	6.34	2.46	4.11	4.89	6.81	1.96	4.37	5.3
	7.81	3.41	4.63	5.95	6.54	2.58	4.4	5.18	7.05	2.07	4.48	5.41
	8.02	3.53	4.89	6.24	6.76	2.72	4.52	5.33	7.27	2.26	4.61	5.6
A20/β-actin												
	0.52	0.23	0.31	0.40	0.43	0.17	0.29	0.35	0.46	0.14	0.30	0.37
	0.53	0.24	0.31	0.41	0.44	0.18	0.31	0.36	0.48	0.14	0.30	0.37
	0.53	0.24	0.32	0.42	0.45	0.19	0.31	0.36	0.49	0.15	0.31	0.38
Mean	0.53	0.24	0.31	0.41	0.44	0.18	0.30	0.36	0.48	0.14	0.30	0.37
Mean ± SD	0.53±0	0.24±0.01	0.31±0.01	0.41±0.01	0.44±0.01	0.18±0.01	0.3±0.01	0.36±0.01	0.48±0.01	0.14±0.01	0.3±0	0.37±0.01

加样孔	1	2	3	4	5	6	7	8	9	10	11	12
样品名称	1-4号	2-23号	3-48号	4-28号	1-6号	2-89号	3-69号	4-47号	1-7号	2-67号	3-13号	4-37号
p-NF-κB p65												
	4.27	13.47	11.1	9.45	6.05	10.28	8.26	7.11	6.82	11.53	9.39	7.8
	4.46	13.72	11.35	9.69	6.23	10.49	8.47	7.34	6.99	11.8	9.49	7.92
	4.6	13.81	11.58	10.03	6.49	10.6	8.69	7.44	7.23	11.91	9.82	8.23
NF-κB p65												

	14.33	14.92	14.81	13.59	13.34	13.18	13.26	13.44	13.55	13.33	13.92	13.11
	14.58	15.04	15.03	13.82	13.45	13.37	13.46	13.7	13.73	13.62	14.04	13.36
	14.73	15.35	15.28	14.1	13.55	13.65	13.58	13.74	13.92	13.64	14.29	13.51
β-actin												
	16.116	15.867	15.865	15.758	15.795	15.225	15.45	15.251	15.537	15.36	15.425	15.794
	16.972	16.72	16.762	16.6	16.655	15.939	16.319	16.051	16.35	16.186	16.426	16.673
	17.432	17.144	17.183	17.045	17.08	16.444	16.716	16.465	16.805	16.615	16.852	17.056
NF-κB p65/β-actin	0.89	0.94	0.93	0.86	0.84	0.87	0.86	0.88	0.87	0.87	0.90	0.83
	0.86	0.90	0.90	0.83	0.81	0.84	0.82	0.85	0.84	0.84	0.85	0.80
	0.84	0.90	0.89	0.83	0.79	0.83	0.81	0.83	0.83	0.82	0.85	0.79
Mean	0.86	0.91	0.91	0.84	0.82	0.84	0.83	0.86	0.85	0.84	0.87	0.81
p-NF-κB p65/β-actin	0.26	0.85	0.70	0.60	0.38	0.68	0.53	0.47	0.44	0.75	0.61	0.49
	0.26	0.82	0.68	0.58	0.37	0.66	0.52	0.46	0.43	0.73	0.58	0.48
	0.26	0.81	0.67	0.59	0.38	0.64	0.52	0.45	0.43	0.72	0.58	0.48
Mean	0.26	0.83	0.68	0.59	0.38	0.66	0.52	0.46	0.43	0.73	0.59	0.48
p-NF-κB p65/NF-κB p65												
	0.30	0.90	0.75	0.70	0.45	0.78	0.62	0.53	0.50	0.86	0.67	0.59
	0.31	0.91	0.76	0.70	0.46	0.78	0.63	0.54	0.51	0.87	0.68	0.59
	0.31	0.90	0.76	0.71	0.48	0.78	0.64	0.54	0.52	0.87	0.69	0.61
Mean ± SD	0.31±0.01	0.9±0.01	0.75±0	0.7±0.01	0.47±0.01	0.78±0	0.63±0.01	0.54±0.01	0.51±0.01	0.87±0	0.68±0.01	0.6±0.01

加样孔	1	2	3	4	5	6	7	8	9	10	11	12
样品名称	1-4号	2-23号	3-48号	4-28号	1-6号	2-89号	3-69号	4-47号	1-7号	2-67号	3-13号	4-37号
p-IκBα												
	1.21	4.87	3.84	3.11	2.3	5.84	4.1	3.18	1.42	4.73	3.65	2.51
	1.33	4.99	3.99	3.09	2.51	6.12	4.36	3.41	1.62	4.89	3.83	2.79
	1.19	4.69	4.1	3.21	2.73	6.36	4.63	3.6	1.91	5.08	4.03	3.01
Mean	1.24	4.85	3.98	3.14	2.51	6.11	4.36	3.40	1.65	4.90	3.84	2.77
IκBα												
	9.16	3.1	6.22	7.51	7.76	3.74	4.84	6.3	9.8	3.19	4	4.13
	9.4	2.89	6.37	7.74	7.93	3.85	5.03	6.59	9.95	3.62	4.26	4.43
	9.54	2.77	6.6	7.99	8.14	4.01	5.26	6.85	10.19	3.72	4.48	4.71

β-actin												
	14.29	15.3	14.02	15.77	14.26	14.55	14.23	14.99	14.05	14.11	14.29	14.1
	14.5	15.44	14.32	15.93	14.53	14.85	14.4	15.13	14.24	14.21	14.52	13.88
	14.79	15.7	14.58	16.2	14.7	15.14	14.67	15.27	14.54	14.47	14.68	13.61
p-IκBα/β-actin	0.08	0.32	0.27	0.20	0.16	0.40	0.29	0.21	0.10	0.34	0.26	0.18
	0.09	0.32	0.28	0.19	0.17	0.41	0.30	0.23	0.11	0.34	0.26	0.20
	0.08	0.30	0.28	0.20	0.19	0.42	0.32	0.24	0.13	0.35	0.27	0.22
Mean	0.09	0.31	0.28	0.20	0.17	0.41	0.30	0.22	0.12	0.34	0.26	0.20
IκBα/β-actin	0.64	0.20	0.44	0.48	0.54	0.26	0.34	0.42	0.70	0.23	0.28	0.29
	0.65	0.19	0.44	0.49	0.55	0.26	0.35	0.44	0.70	0.25	0.29	0.32
	0.65	0.18	0.45	0.49	0.55	0.26	0.36	0.45	0.70	0.26	0.31	0.35
Mean	0.64	0.19	0.45	0.49	0.55	0.26	0.35	0.43	0.70	0.25	0.29	0.32
p-IκBα/IκBα												
	0.13	1.57	0.62	0.41	0.30	1.56	0.85	0.50	0.14	1.48	0.91	0.61
	0.14	1.73	0.63	0.40	0.32	1.59	0.87	0.52	0.16	1.35	0.90	0.63
	0.12	1.69	0.62	0.40	0.34	1.59	0.88	0.53	0.19	1.37	0.90	0.64
均值	0.13	1.66	0.62	0.41	0.32	1.58	0.86	0.52	0.17	1.40	0.90	0.63
Mean ± SD	0.13±0.01	1.66±0.08	0.62±0	0.41±0.01	0.32±0.02	1.58±0.02	0.86±0.02	0.52±0.01	0.17±0.02	1.4±0.07	0.9±0.01	0.63±0.02

加样孔	1	2	3	4	5	6	7	8	9	10	11	12
样品名称	1-5号	2-89号	4-123号	5-126号	1-12号	2-125号	4-15号	5-79号	1-7号	2-67号	4-78号	5-57号
NF-κB p65												
	30.15	284.77	114.23	57.75	59.81	316.77	156.41	104.08	42.32	276.48	118.33	125.72
	31.64	284.62	115.77	58.15	60.39	316.76	156.41	105.71	43.58	277.30	117.86	127.74
	28.54	285.50	112.82	57.68	59.81	316.76	157.67	106.80	43.04	277.65	120.98	125.18
Histone H3												
	265.51	210.93	262.59	202.95	197.01	188.31	209.19	236.24	220.15	191.97	190.83	231.44
	265.33	212.11	261.94	203.49	196.33	188.21	208.51	237.86	219.63	191.78	190.29	232.27
	265.50	210.89	260.12	203.08	198.45	188.31	209.54	235.24	220.52	191.55	191.66	230.87
NF-κB p65<br>/Histone H3												
	0.11	1.35	0.44	0.28	0.30	1.68	0.75	0.44	0.19	1.44	0.62	0.54
	0.12	1.34	0.44	0.29	0.31	1.68	0.75	0.44	0.20	1.45	0.62	0.55
	0.11	1.35	0.43	0.28	0.30	1.68	0.75	0.45	0.20	1.45	0.63	0.54
Mean	0.11	1.35	0.44	0.28	0.30	1.68	0.75	0.45	0.20	1.45	0.62	0.55
Mean ± SD	0.11±0.01	1.35±0.01	0.44±0	0.28±0	0.3±0	1.68±0	0.75±0	0.45±0.01	0.2±0	1.45±0	0.62±0.01	0.55±0
